# Intravenous Ibuprofen for Treatment of Post-Operative Pain: A Multicenter, Double Blind, Placebo-Controlled, Randomized Clinical Trial

**DOI:** 10.1371/journal.pone.0154004

**Published:** 2016-05-06

**Authors:** Andrea Gago Martínez, Blanca Escontrela Rodriguez, Antonio Planas Roca, Alberto Martínez Ruiz

**Affiliations:** 1 Department of Anesthesia, Resuscitation and Pain Therapy, Cruces University Hospital, Barakaldo, Vizkaya, Spain; 2 Department of Anesthesia, Resuscitation and Pain Therapy, The Princess University Hospital, Madrid, Spain; Cardiff University, UNITED KINGDOM

## Abstract

**Background:**

Non-steroidal anti-inflammatory drugs are often used as components of multimodal therapy for postoperative pain management, but their use is currently limited by its side effects. The specific objective of this study was to evaluate the efficacy and safety of a new formulation of intravenous (IV) ibuprofen for the management of postoperative pain in a European population.

**Methods and Findings:**

A total of 206 patients from both abdominal and orthopedic surgery, were randomly assigned in 1:1 ratio to receive 800 mg IV-ibuprofen or placebo every 6 hours; all patients had morphine access through a patient controlled analgesia pump. The primary outcome measure was median morphine consumption within the first 24 hours following surgery. The mean±SEM of morphine requirements was reduced from 29,8±5,25 mg to 14,22±3,23 mg (p = 0,015) and resulted in a decrease in pain at rest (p = 0,02) measured by Visual Analog Scale (VAS) from mean±SEM 3.34±0,35 to 0.86±0.24, and also in pain during movement (p = 0,02) from 4.32±0,36 to 1.90±0,30 in the ibuprofen treatment arm; while in the placebo group VAS score at rest ranged from 4.68±0,40 to 2.12±0,42 and during movement from 5.66±0,42 to 3.38±0,44. Similar treatment-emergent adverse events occurred across both study groups and there was no difference in the overall incidence of these events.

**Conclusions:**

Perioperative administration of IV-Ibuprofen 800 mg every 6 hours in abdominal surgery patient’s decreases morphine requirements and pain score. Furthermore IV-Ibuprofen was safe and well tolerate. Consequently we consider appropriate that protocols for management of postoperative pain include IV-Ibuprofen 800 mg every 6 hours as an option to offer patients an analgesic benefit while reducing the potentially risks associated with morphine consumption.

**Trial Registration:**

EU Clinical Trials Register 2011-005007-33

## Introduction

Nowadays there is no place to debate the need of assertive treatment of acute postoperative pain [1–4]. We all agree on the importance of multimodal treatment of perioperative pain, since it has direct effects on the outcome of patient recovery [2–5]. Despite this knowledge, acute postoperative pain still is a significant problem that occurs between 60 to 80% of patient’s according the different series reported and it often remains undertreated [6–8]. Opioid analgesics are the mainstay of acute pain treatment in the inpatient setting [9]. However they provide no benefits to the underlying pathophysiology of the process. Adjuvant agents for pain treatment including non-steroidal anti-inflammatory drugs (NSAID) should be used in combination with opioids [1,3,10]. Moderate to severe acute postoperative pain should be treated with a multimodal therapy and a preemptive analgesia approach that may help mitigate the side effects of the opioid agents by reducing the total dose required [3,4,11,12,13]. There are a few NSAID available for intravenous use in Spain (Ketorolac, Dexketoprofen) being their usage limited by the side effects and prolonged periods of treatment are not recommended. Ibuprofen is a nonspecific inhibitor of cyclooxygenases (COX) enzymes, the inhibition rate of COX-1 to COX 2 of Ibuprofen is 2,5:1 which implies a low risk of bleeding or gastrointestinal problems, while other NSAID as for example ketorolac have a ratio of COX-1 to COX-2 inhibition of 330:1, reason for a high risk of side effects and it is therefore its use is controversial in most of perioperative settings [14]. Oral ibuprofen is a commonly used NSAID with antipyretic, anti-inflammatory and analgesic properties, but this presentation is not appropriate in perioperative settings, then its use has been previously limited by the lack of a commercially available parenteral formulation in our country. Until now three surgical studies were conducted to assess the use of intravenous (IV) ibuprofen for the treatment of postoperative pain (outside Europe). The first one evaluates the efficacy and safety in patients undergoing either orthopaedic or abdominal surgery (mostly corresponding to abdominal hysterectomies) [9], the second one [8] evaluates the efficacy in patients undergoing orthopaedic surgery and the third one [13] evaluates the patients undergoing abdominal hysterectomy. These studies have demonstrated beneficial effects of perioperative administration of IV ibuprofen for moderate to severe postoperative pain, and set the stage for further investigations and clinical uses [14]. The present phase III clinical trial is part of our effort to expand the availability of treatment options for multimodal analgesic therapy in Europe. The specific objective of this study was to evaluate the efficacy of a new formulation of IV-Ibuprofen compared to placebo for the management of postoperative pain in abdominal and orthopaedic surgery. The primary efficacy endpoint was the reduction in total morphine use during the first 24 hours post-surgery as compared to placebo. The secondary objective was to evaluate the tolerability and safety profile of intravenous administration of ibuprofen.

## Methods

### Trial Design

This was a phase III, multi-center, randomized [1:1], double-blind, parallel groups, placebo-controlled trial, designed to evaluate the safety and efficacy of IV-Ibuprofen for the treatment of pain in post-operative elective abdominal or orthopedic surgery in adult patients, with an anticipated need for post-operative hospitalization and IV-Morphine analgesia for at least 24 hours. The study followed the principles of Helsinki Declaration and was approved by institutional review boards and independent ethics committees at all participating sites: Hospital Universitario Cruces, Hospital Universitario Arnau de Vilanova de Lleida, Clínica Universitaria de Navarra, Hospital Universitario de la Princesa, Hospital Clínico Universitario, Complejo Hospitalario Universitario A Coruña, Fundación Jiménez Díaz, Hospital Móstoles and Complejo Hospitalario Universitario de Vigo (URL: https://www.clinicaltrialsregister.eu, EudraCT Number: 2011-005007-33, Sponsor Protocol Number: BIBEC02).

Patients, who met all of the inclusion criteria, did not meet any exclusion criteria (Table 1) and had signed the Informed Consent Form (ICF) were randomized in a 1:1 ratio to receive an infusion of either 800mg/200ml of IV-Ibuprofen or placebo, every 6 hours, over a 15 minute period. Randomized stratification was used according to baseline patient characteristics, type of surgery and centre. In order to avoid any bias and maintain the integrity of the data, the trial was double blind (Pivotal S.L generated the randomization sequence, managed through e-CRF). Adequate drug labelling warranted blinding of both, the patient and the research study staff. The study design took into account previous pain management trials, current concepts related to phase III clinical trials and guidelines regarding clinical trials linked to pain, including “Note for guidance on clinical investigation of medicinal products for treatment of nociceptive pain; CPMP/EWP/612/00”. The selection of the dose and frequency of Ibuprofen administration as well as the choice of placebo control was based on phase I clinical trial results (BIBEC01, EudraCT NCT: 2011-002148-29), conducted on 24 healthy volunteers and on results of previous clinical studies of other intravenous forms of ibuprofen for the management of post-operative pain in hospitalized patients [8,9,13].

**Table 1:** Eligibility criteria for participants.

**Inclusion Criteria:**
1. Men or women between 18 and 80 years old.
2. Scheduled for elective single surgical site orthopedic surgery (hip or knee joint replacement, crossed ligaments, spine or shoulder surgery), or abdominal surgery (inguinal hernia, cholecystectomy, eventration or hiatus hernia).
3. Scheduled for general anesthesia or regional anesthesia without residual analgesia after surgery.
4. Anticipated need for postoperative narcotic analgesia administered by patient controlled analgesia.
5. Anticipated hospital stays for at least 24 hours.
6. Written informed consent for participating in this study.
**Exclusion Criteria:**
1. Use of NSAID within 12 hours prior to the first planned dose.
2. Taking oral anticoagulants, lithium, ACE inhibitors, furosemide or aspirin.
3. Anemia (hemoglobin <10 g/dl) and/or history or evidence of asthma or heart failure.
4. History of allergy or hypersensitivity to any component of intravenous ibuprofen, aspirin or aspirin related products, NSAID or COX-2 inhibitors.
5. Pregnant or nursing (pregnancy test should be made to fertile women. Those women with 12 months of amenorrhea were considered menopause women).
6. Weight less than 40 kg.
7. History of severe head trauma that required hospitalization, intracranial surgery or stroke within the previous 30 days, or any history of intracranial arteriovenous malformation, cerebral aneurism or CNS mass lesion.
8. History of congenital bleeding diathesis or any active clinically significant bleeding or underlying platelet dysfunction.
9. Gastrointestinal bleeding that required medical intervention.
10. History of Peptic ulcer or Intestinal Inflammatory disease.
11. Severe cardiac insufficiency and/or ischemic cardiomyopathy.
12. Platelet count less than 80.000.
13. Pre-existing dependence on narcotics or receiving chronic treatment with opioids.
14. Severe renal failure (calculated creatinine clearance < 60 ml/min).
15. Liver failure, ALAT or ASAT >3 times upper limit of normality or total bilirubin >2 mg/dl.
16. Diagnosed of Bowel Inflammatory Disease.
17. Not able to understand the requirements of the study, or to abide by the study restrictions or to return for the required assessments.

ACE = angiotensin-converting enzyme; NSAID = non-steroidal anti-inflammatory drug; COX-2 = cyclooxygenase 2; CNS = central nervous system; ALAT: Alanine Amino Transferase; ASAT: Aspartate Amino Transferase.

Regarding the patient inclusion criteria we must emphasize that two important amendments to the original protocol were performed in order to improve the enrolment rate above all on the arm of orthopaedic surgery: a) the first amendment added new surgical indications to the inclusion criteria: spinal, shoulder, ligaments, evisceration and hiatus hernia; b) the second amendment allowed the enrolment of patients undergoing regional anaesthesia without residual analgesic effect after surgery and extended the screening period from 14 to 30 days prior to surgery. The latter modification also includes changes in the analysis of the results (interim analysis) that will be explained with the selection of sample size.

### Participants and Interventions

The trial was performed at the inpatient setting of a total of nine Spanish hospitals: Hospital Universitario Cruces (Bilbao), Hospital Universitario Arnau de Vilanova de Lleida (Lleida), Clínica Universitaria de Navarra (Pamplona), Hospital Universitario de la Princesa (Madrid), Hospital Clínico Universitario (Madrid), Complejo Hospitalario Universitario A Coruña (CHUAC) (Coruña), Fundación Jiménez Díaz (Madrid), Hospital Móstoles (Madrid) and Complejo Hospitalario Universitario de Vigo (Vigo). Enrolment was open to adults between 18 to 80 years who were scheduled for elective single surgical site orthopaedic surgery (hip or knee joint replacement, coursed ligaments, column or shoulder surgery), or abdominal surgery (inguinal hernia, cholecystectomy, eventration or hiatus hernia) (Table 1), and who were expected to require postoperative hospitalization and intravenous morphine analgesia for at least 24 hours. Participating patients had to have signed the informed consent during the screening period and had to be able to provide a reliable self-report of pain. We shall point out that the study treatment had to be discontinued: if the patient received either narcotic pain medication (other than morphine) or non-narcotic pain medication (including another NSAID), Paracetamol or Metamizol, or was able to tolerate oral pain medication; the pain was resolved; there was a loss of intravenous access; the patient was discharged from the hospital; or the patient decided to withdraw for any reason and at any time.

**Fig 1:**
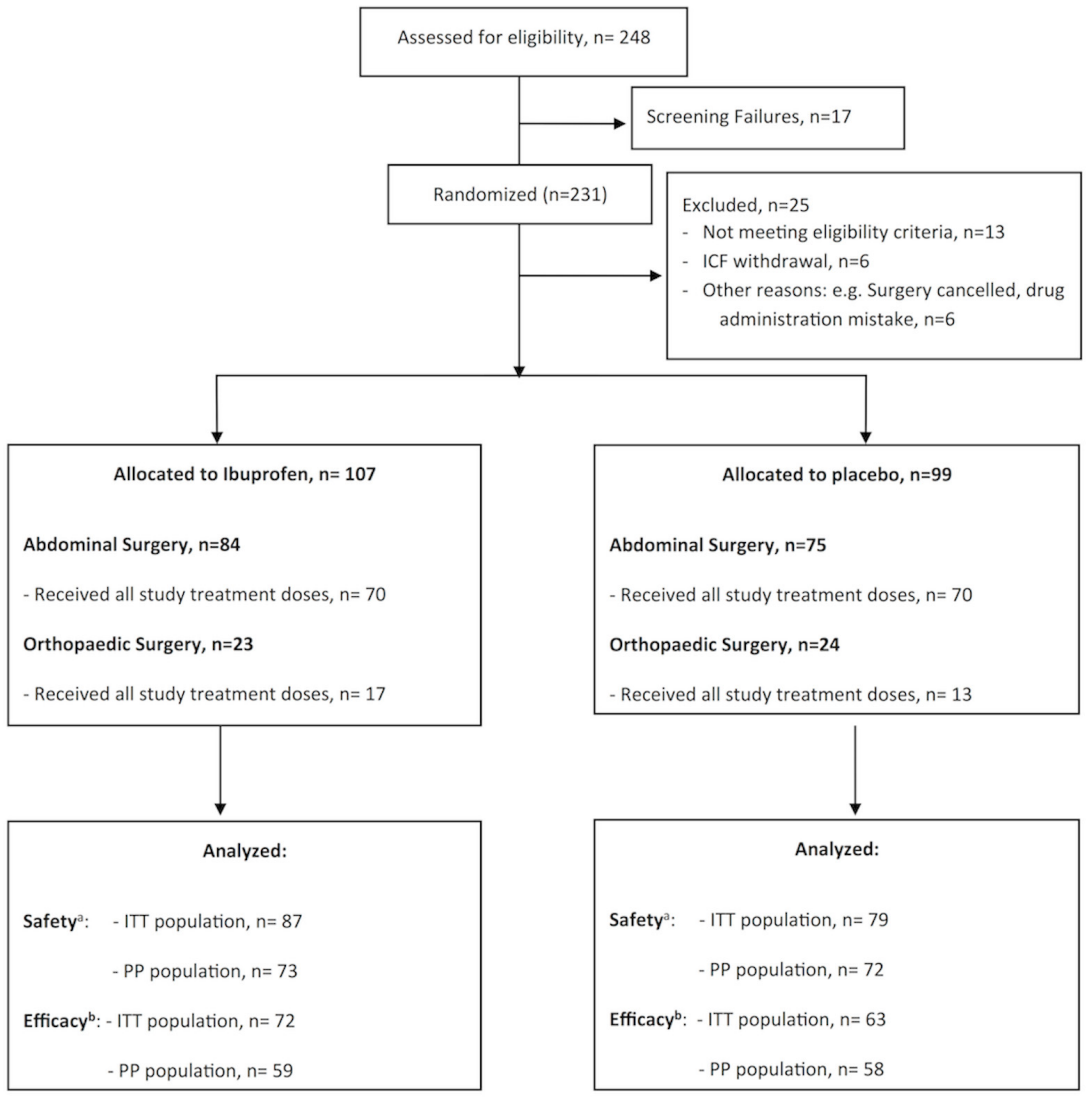
Distribution of patients randomized to receive IV-Ibuprofen or placebo for the management of postoperative pain. ICF: Informed Consent Form. ^a^ Safety: all patients who received at least one dose of IV-Ibuprofen or placebo (patients from both type of surgery abdominal and orthopedic) ^b^ Efficacy: all patients from abdominal surgery who received at least 4 doses of IV ibuprofen or placebo within 30 minutes of the scheduled administration time. Reasons for exclusion from the efficacy population included adverse events, noncompliance, and lack of efficacy, among others that resulted in the failure to complete the study protocol. Excluded from Efficacy analysis all patients from Orthopaedic surgery (because of the low rate of enrolment of patients due to the type of anaesthetic technique used mostly regional type): Ibuprofen arm: ITT n = 15; PP n = 14. Placebo group: ITT n = 16; PP n = 14. ^c^ ITT = Intention to Treat: population consisted of all patients who were both randomized and treated. ^d^ PP = Per Protocol: population consisted of all patients from the ITT population who had received the study treatment for at least 24 hours.

**Fig 2:**
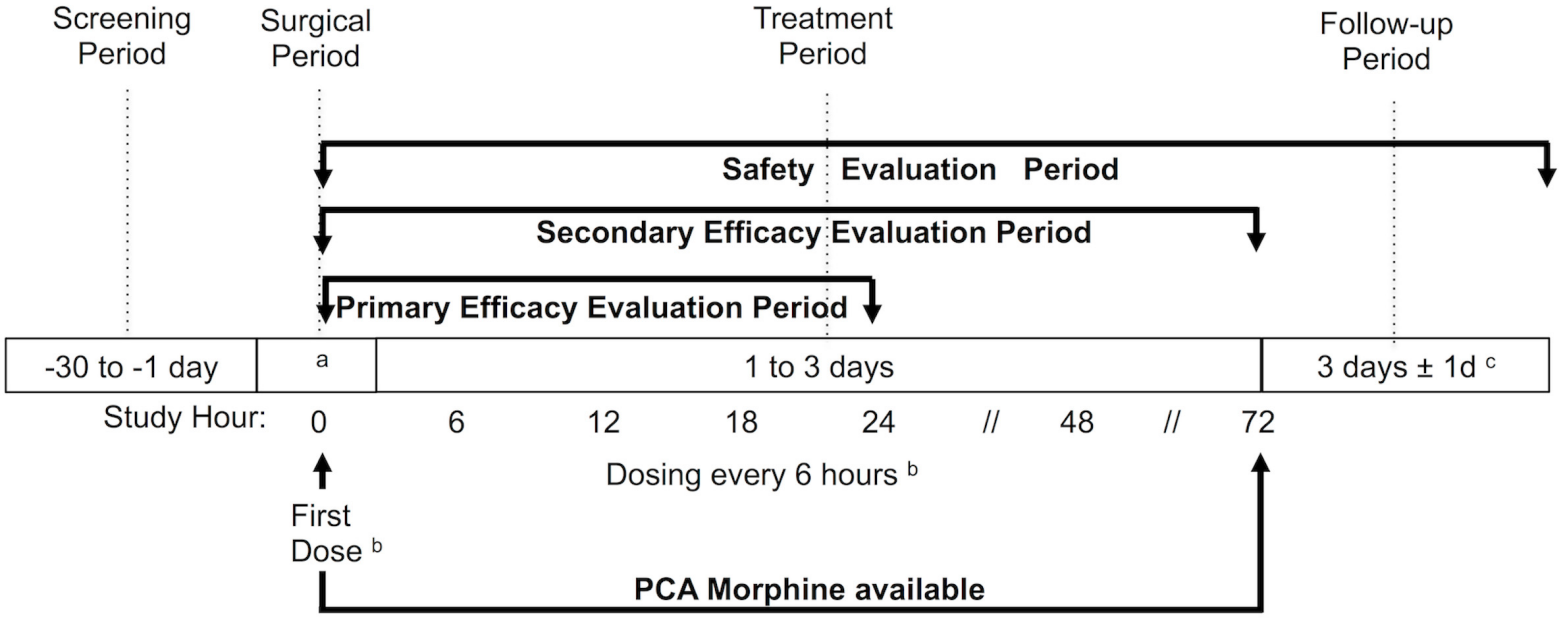
Study Timeline. ^a^ Site closure. ^b^ IV-Ibuprofen 800 mg or saline placebo, up to 24 hours for abdominal surgery and up to 72 hours for orthopedic surgery. ^c^ Counted since the last dose administered.

Patients were randomized to receive either 800 mg/200 ml (4 mg/ml) of IV-Ibuprofen or placebo (saline solution 200 ml) (Fig 1). The first dose of the study drug was administered upon initiation of skin closure, subsequent doses of study drug were to be administered every 6 hours (the dose was infused in 15 minutes), the clinical trial treatment duration was 24 hours for abdominal surgery, 48 hours for hip, shoulder and ligaments surgery and 72 hours for knee and spine surgery (Fig 2).

At the time of discharge from the operating room, all patients had access to intravenous morphine through patient controlled analgesia (PCA), with an established dose of 1 mg per bolus dose with a lock-out time of 5 min and maximum dose limits of 10 mg in an hour and 30 mg in 4 hours. If adequate pain control was not achieved then additional morphine could be administered and registered by the patient's physician. However, basal infusions of morphine were not allowed.

For patients who received general anesthesia, the anesthetic drug was either propofol or a member of flurane family and the analgesic used for intraoperative narcotic maintenance was fentanyl. The Intraoperative use of any other analgesics including other NSAIDs and/or catheter-based regional anesthesia techniques was prohibited.

The date range for recruitment and monitoring of patients was from March 11, 2012 to June 02, 2014.

### Efficacy Assessment

The primary efficacy endpoint was a reduction in the requirement for morphine during the first 24 hours after surgery comparing total morphine usage of the active arm versus the placebo arm. Secondary efficacy endpoints included: reduction in pain intensity at rest and during movement, reduction in morphine consumption during the first 48 and 72 hours post-surgery, time to first subsequent narcotic analgesia for breakthrough pain (or time to treatment failure), reduction in the number of doses of morphine and in the number of attempts of PCA dosing, less nocturnal awakening due to pain and the score on the Ramsay-Hunt sedation scale. Pain intensity was measured by patient self-assessment both at rest and during movement with the Visual Analogue Scale (VAS) at 1, 3, 6, 12, 18 and 24 hours following surgery, combined with Ramsay-Hunt sedation scale. The VAS was presented as a 10 cm horizontal line anchored by the words "no pain" (corresponding to zero) and "worst possible pain" (corresponding to 10). The patient was taught to mark the point on the line that they felt represented their perception of their current pain. The values were scored as mild pain (from 1 to 4), moderate pain (from 5 to 6) and severe pain (from 7 to 10) [15].

### Safety Assessments

At the beginning of the trial a complete physical examination of all participants was performed, baseline vital signs were recorded, blood samples were drawn for clinical chemistry (included: sodium, potassium, chloride, glucose, blood urea nitrogen, creatinine, total bilirubin, albumin, total protein, aspartate aminotransferase, alanine aminotransferase), haematology (haematocrit, haemoglobin, platelet count, white blood cell count) and coagulation analysis (activated partial thromboplastin time, prothrombin time and INR); concomitant medications were reviewed and recorded for each patient. Tolerability and safety profile of 800 mg IV-Ibuprofen versus placebo, both infused every 6 hours, was evaluated through a comparison of treatment emergent adverse events (AE) [by MedDRA System Organ Class (SOC)], local reaction due to intravenous infusion (pain, erythema, induration) as well as vital signs, clinical chemistry values, haematology and coagulation values. Researchers were responsible for identifying and promptly reporting any AE that occurred during the study period and to grade the event by intensity from 1 to 3 [according to NCI Common Terminology Criteria for Adverse Events (CTCAE) version 4], recording action taken with the study drug, causality of the study drug, required treatment, outcome and time of resolution. Vital signs and laboratory results were monitored closely to investigate the effects of the treatment, including renal, liver and haematology values. Vital signs were assessed at baseline and at 1, 3, 6 hours after the initial dose and every 6 hours thereafter concomitant with study drug administration until the final dose and during the follow- up visit (within 2 to 4 days after the last dose of the study drug treatment). Routine laboratory tests (chemistry, haematology and coagulation) were done during screening as baseline, within 24 hours after the first dose for abdominal surgery, at 48 hours after the first dose for patients with hip surgery, shoulder, spine and ligaments and at 72 hours after the first dose for patients with knee or column surgery, and at the follow up visit. Adverse event monitoring continued through day of the follow-up visit, in the event that a patient could not attend the follow-up visit, safety assessments were conducted by telephone.

### Statistical Analysis

Sample size determination was based on previous IV-ibuprofen studies and on literature review [8,9,13]. It was projected that the morphine requirement in the placebo group would be approximately 55 mg and that the ibuprofen treatment would reduce it to around 45 mg. Accepting an alpha level of 0.05 and a beta risk of 0.2 in a two-sided test, 143 subjects were deemed necessary in each group to recognize as statistically significant difference greater than or equal to 10 mg. The standard deviation was assumed to be 30. Considering a dropout rate of 10–12%, a total of 320 patients were to be included, 160 in each treatment group. If half of the patients correspond to each type of surgery (orthopaedic and abdominal surgery) the power to evaluate differences in each group would be 55%. An interim analysis (according to the second amendments to the original protocol) was performed once half of the planned sample (160 patients) had completed the study, in order to evaluate whether or not it was appropriate to continue the trial, and/or to increase or decrease the sample size according to the differences found between the two treatment groups. Statisticians who did not participate in the evaluation of patients carried out this analysis, and the results were not disclosed to the study researchers. For this statistical comparison was considered a significance level of p <0.015. Recruitment was to be stopped if significant differences for the two subgroups (abdominal surgery and orthopaedic surgery) were reached. If not, sample size was to be calculated based on the data obtained. All recruiting hospitals had the same case report forms, with an instruction manual providing detailed instructions for each data item, and the research team were trained on the data collection procedures required.

For statistical analyses of the efficacy endpoints two populations were defined: the Intention To Treat (ITT) population consisting of all patients who were both randomized and treated, (only patients included in the ITT population from the interim analysis were included in the final ITT population); and the Per Protocol (PP) population consisting of all patients from the aforementioned ITT population who had received the study treatment for at least 24 hours. The safety analysis was performed based on all the subjects who had received at least one dose of the study medication. Continuous variables were summarized using the mean, standard error of the mean (SEM), median, Q1, Q3, minimum and maximum with the total number of patients contributing values. The Shapiro-Wilk test was used to assess if the continuous measure followed a Normal distribution. Categorical variables were summarized in contingency tables by presenting the absolute and relative frequencies of patients in each category. CI 95% for categorical variables was included in the efficacy analysis. The association between continuous variables and the treatment group were analysed following the T-Student test if the variable followed Normal distribution and the Wilcoxon test if it did not. The association between categorical variables and the treatment were analysed following the Chi-square test or F- Fisher test as applicable. The dependent variable was the evaluated measure and the independent variables were the treatment and the visit or evaluation moment. To analyse the time until patients presented a determined event, a Kaplan-Meier model stratified by treatment group was applied. Comparisons between treatment groups were performed through the Log-Rank test. Statistical tables were created using the SAS version 9.3 statistical packages. A bilateral risk of α = 0.015 was fixed for all analyses performed. Adverse events were coded using MedDRA system organ classifications, and the adverse events were also coded as serious or non-serious using FDA definitions.

## Results

A total of 206 patients were enrolled at nine surgical centres in Spain, Fig 1 shows the distribution. The demographics and characteristics of the study population are shown in Table 2. Study treatment arms were well balanced regarding patient characteristics at baseline with no differences between both groups. Besides, there were no differences in those characteristics between ITT and PP populations.

**Table 2:** Summary of Baseline Demographics and Patient Characteristics.

Patients characteristics	ITT Population			PP Population		
	Placebo + PCA Morphine (n = 79)	IV-Ibuprofen + PCA Morphine (n = 87)	p-value	Placebo + PCA Morphine (n = 72)	IV-Ibuprofen +PCA Morphine (n = 73)	p-value
Age (years)			0.8			0.7
Mean ± SD	53.03 ± 14.45	53.49 ± 12.58		51.93 ± 14.30	52.56 ± 12.47	
Gender			0.7			0.6
Male, n (%)	53.03 ± 14.45	53.49 ± 12.58		32 (44.44)	35 (47.95)	
Female, n (%)	53.03 ± 14.45	53.49 ± 12.58		40 (55.56)	38 (52.05)	
Body Mass Index (Kg/m^2^)			0.6			0.4
Mean ± SD	27.21 ± 4.47	27.24 ± 3.70		27.10 ± 0.41	27.32 ± 3.83	
Temperature (°C)			0.4			0.3
Mean ± SD	36.35 ± 0.43	36.24 ± 0.53		36.37 ± 0.41	36.25 ± 0.51	
Type of surgery			0.6			0.9
Orthopedic, n (%)	16 (20.25)	15 (17.24)		14 (19.44)	14 (19.18)	
Abdominal, n (%)	63 (79.75)	72 (86.74)		58 (80.56)	59 (82.82)	
Abdominal surgery			0.6			0.6
Cholecystectomy, n (%)	57 (72.15)	61 (70.11)		53 (73.61)	50 (68.49)	
Eventration, n (%)	0 (0.00)	1 (1.15)		0 (0.00)	1 (1.37)	
Inguinal Herniorrhaphy, n (%)	6 (7.59)	10 (11.49)		5 (6.94)	8 (10.96)	
Orthopedic surgery			0.4			0.4
Knee, n (%)	7 (8.86)	3 (3.45)		5 (6.94)	2 (2.74)	
Hip, n (%)	6 (7.59)	9 (10.34)		6 (8.33)	9 (12.33)	
Spine, n (%)	1 (1.27)	0 (0.00)		1 (1.39)	0 (0.00)	
Instrumented Arthrodesis, n (%)	2 (2.53)	3 (3.45)		2 (2.78)	3 (4.11)	

### Efficacy Results

Because the enrolment of orthopaedic surgery patients was very low (n = 31) (due to most of these surgeries were performed under regional anaesthesia exclusively, and not as a result of factors related to the clinical trial), while the vast majority of the patients were in the abdominal surgery group (n = 135) where significant differences between the two treatment arms were found, the sponsor decided to stop the enrolment of patients. Therefore, the analysis was limited to abdominal surgery patients, 72 of which were treated with ibuprofen while 63 were treated with placebo.

The primary endpoint was to evaluate the total morphine usage in the first 24 hours post-surgery. Table 3 details for both the ITT (n = 166) and PP (n = 145) populations the mean±SEM amount of total morphine consumption, number of doses of morphine administered and the number of attempts of morphine consumption via PCA. The mean amount of morphine consumption within the first 24 hours after surgery was considerably lower in the patients treated with ibuprofen than in patients who received placebo. As we can also observe in Table 3, the difference in favour of ibuprofen treated patients regarding the numbers of morphine doses administered and the numbers of PCA morphine dosing attempts.

**Table 3:** Data related to Morphine consumption during the first 24 hours post-surgery.

	ITT Population			PP Population		
	Placebo (n = 63)	Ibuprofen (n = 72)	p-value	Placebo (n = 58)	Ibuprofen (n = 59)	p-value
**Amount of Morphine (mg)**						
**N**	61	67		56	57	
**Missing data**^a^	2	5		2	2	
**Mean ± SEM**	32.18 ± 5.30	17.36 ± 3.59	0.01	29.88 ± 5.29	14.22 ± 3.23	0.01
**Number of morphine doses (mg)**						
**N**	60	67		55	57	
**Missing data**^a^	3	5		3	2	
**Mean ± SEM**	11.93 ± 1.41	8.28 ± 1.21	< 0.01	11.60 ± 1.34	8.74 ± 1.37	0.02
**Number of attempts of dosing at PCA**						
**N**	60	63		55	53	
**Missing data**^a^	3	9		3	6	
**Mean ± SEM**	19.33 ± 3.71	13.52 ± 2.86	< 0.01	18.09 ± 3.75	13.68 ± 3.12	0.02

^a^ Number of patients missing data.

**Fig 3:**
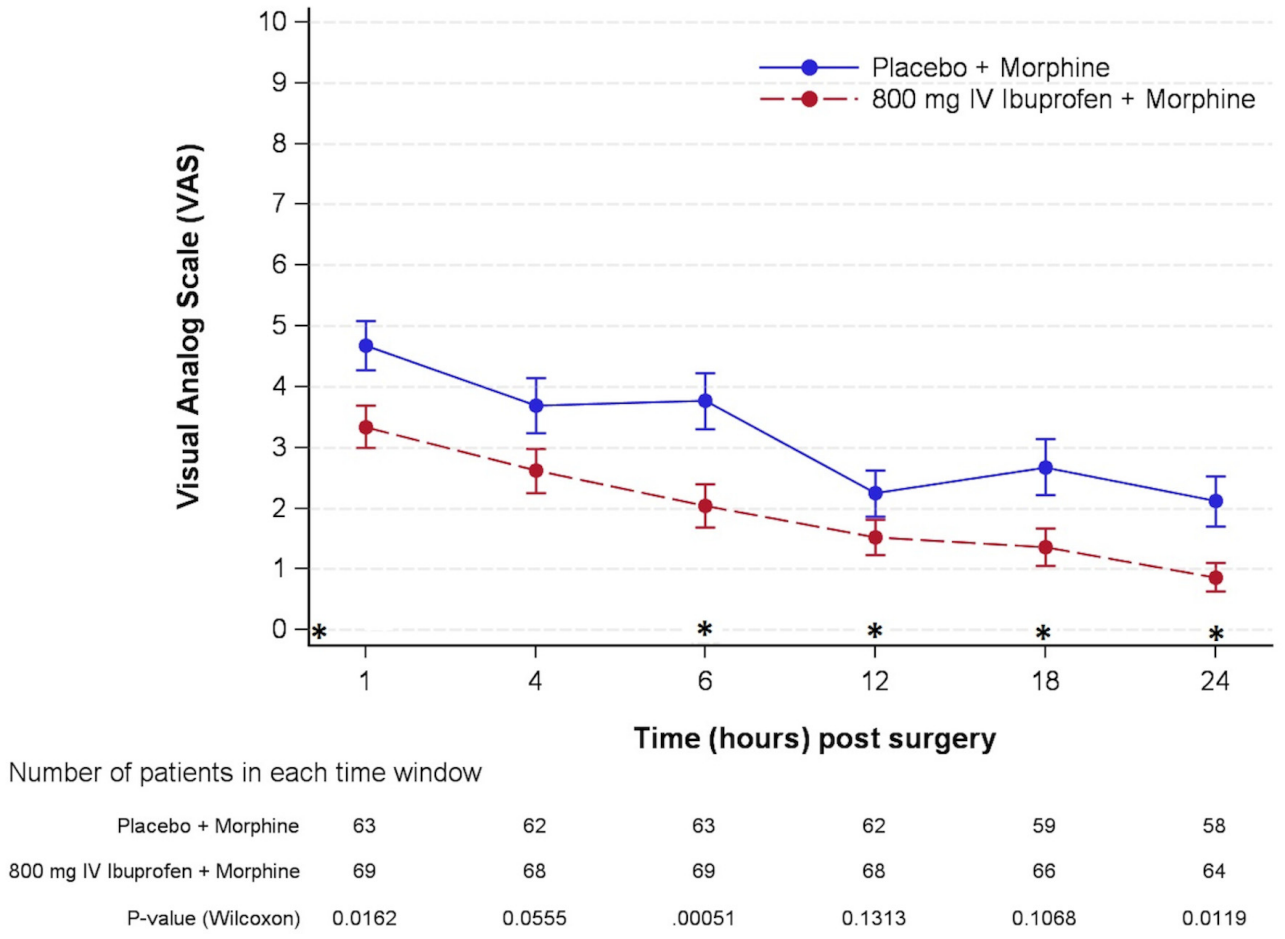
Evolution of postoperative pain at rest. Patients self-reported pain scores during the immediate 24 hours after surgery were plotted for the IV-ibuprofen and placebo treatment groups. Values are mean±SEM. *Treatment doses: First intra-operative administration (T = 0), followed by doses 2, 3, 4 and 5 administered at hours 6, 12, 18 and 24 after surgery.

Pain at rest was better controlled in patients treated with ibuprofen than in patients treated with placebo, (ITT: p< 0.01; PP: p< 0.01). In the ibuprofen arm the mean±SEM pain reduced from 3.34 ± 0.35 to 0.86 ± 0.24 at 24 h. In patients who received placebo pain also reduced from mean±SEM 4.68 ± 0.40 to 2.12 ± 0.42 at 24 h (Fig 3).

**Fig 4:**
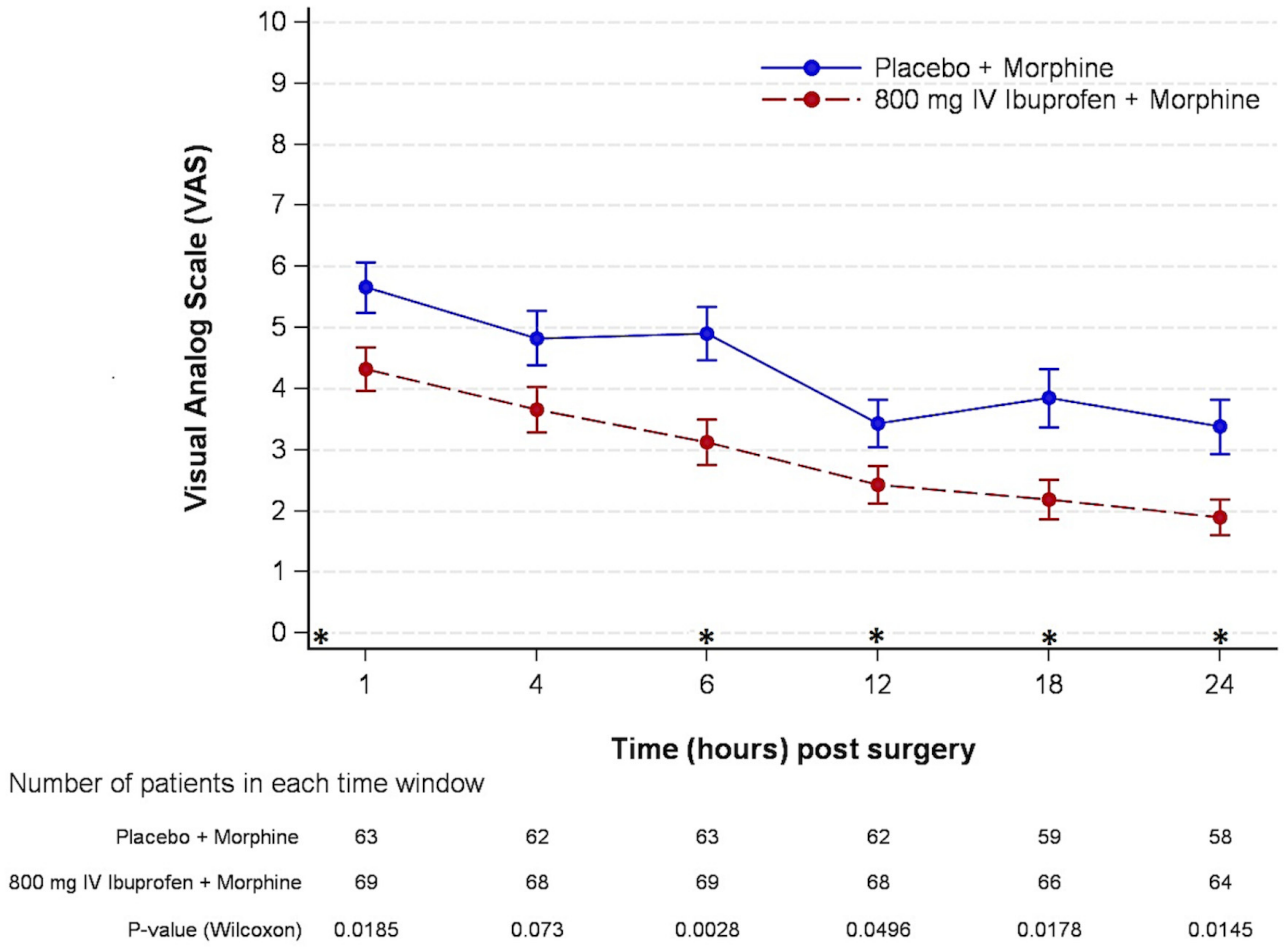
Evolution of postoperative pain during movement. Patients self-reported pain scores during the immediate 24 hours after surgery were plotted for the IV-ibuprofen and placebo treatment groups. Values are mean±SEM. *Treatment doses: First intra-operative administration (T = 0), followed by doses 2, 3, 4 and 5 administered at hours 6, 12, 18 and 24 after surgery.

Pain intensity during movement through the first 24 h was reduced in both treatment arms and was better controlled in ibuprofen-treated patients versus placebo-treated patients (ITT: p = 0.01; PP: p = 0.01). The mean±SEM pain scores decreased from 4.32 ± 0.36 to 1.90 ± 0.30 at 24 hours in the ibuprofen treatment arm, while the corresponding ones for the patients treated with placebo were higher, from 5.56 ± 0.42 to 3.38 ± 0.44 (Fig 4).

Relative to Ramsay-Hunt sedation scale, 1 h after surgery 82.54% and 81.94% of patients treated with placebo and ibuprofen, respectively, were calm, co-operative and communicative. From then up to 24 h the proportion of patients being calm, co-operative and communicative were also very similar in both treatment arms, ranging between 87.54% and 94.44% in the ibuprofen group, and between 88.89% and 96.83% in the placebo group (ITT: p = 0.6; PP: p>0,99).

About the Time to first subsequent narcotic analgesia (or time to treatment failure), within the ITT population, during the study treatment period 18 patients (28.57%) from the placebo arm required treatment with other prohibited narcotics, NSAIDs or analgesics, compared to 8 patients (11.11%) from the ibuprofen arm (p = 0.01). A Kaplan-Meier analysis with Log-Rank test was performed in order to compare the time to treatment failure between both arms. This analysis showed that there was a significantly higher risk for treatment failure in patients treated with placebo [HR = 0.38 (95% CI: 0.16, 0.88); p = 0.01]. Very similar results were obtained in the PP population, in which 16 patients (27.59%) treated with placebo required rescue treatment, compared to 4 patients (6.78%) treated with ibuprofen (p<0.01), the HR was 0.23 [(95% CI: 0.07, 0.69); p<0.01].

Analyses of nocturnal awakenings due to pain during the first 24 hours after surgery, revealed that in the ITT population 7 patients (11.11%) and in PP population 6 patients (10.34%) treated with placebo, suffered nocturnal awakenings. The proportion of patients treated with ibuprofen who experienced nocturnal awakenings during the same time period was lower (ITT: 4 patients, 5.56%; PP: 2 patients, 3.39%). However, the comparisons between both treatment groups were not substantial (ITT: p = 0.3; PP: p = 0.1).

### Safety Results

A total of 206 patients, 107 treated with ibuprofen and 99 treated with placebo, formed the Safety population. Eighty-seven out of 107 patients (81.31%) received all the scheduled ibuprofen doses. Regarding patients treated with placebo, 83 out of 99 patients (83.84%) received all the scheduled placebo doses. A total of 91 Adverse Events (AE) were reported during the study, 45 from patients treated with ibuprofen (42.1%) and 46 from patients treated with placebo (46.5%). In the ibuprofen treatment arm, 27 patients (25.3%) presented at least 1 AE during the study, compared to 33 patients (31.31%) in the placebo group; data are shown in Table 4. Furthermore most of the AEs were mild or moderate, as only 2 patients treated with ibuprofen and 1 patient treated with placebo had grade 3 or Severe AE (SAE) during the course of the trial (Table A in S1 Table). All SAE were assessed as not related to the study treatment.

**Table 4:** Adverse events summary.

	ITT Population (n = 206)			PP Population (n = 182)		
	Ibuprofen (n = 107)	Placebo (n = 99)	p-value	Ibuprofen (n = 91)	Placebo (n = 91)	p-value
Patients with at least one adverse event, n (%)	27 (25.3)	31 (31.31)	0.3	27 (29.67)	30 (32.97)	0.6
Patients with at least one grade 3 adverse events, n (%)	2 (1.87)	1 (1.01)	>0,99	2 (2.2)	1 (1.1)	>0,99
Patients with at least one adverse event that led to permanently treatment discontinuation, n (%)	3 (2.80)	5 (5.05)	0.4	1 (1.1)	5 (5.49)	0.2
Patients with at least one adverse event that the investigator considered related with study medication, n (%)	10 (9.35)	6 (6.06)	0.3	9 (9.89)	5 (5.49)	0.2
Patients with at least one grade 3 adverse events that the investigator considered related with study medication, n (%)	1 (0.93)	0 (0.00)	>0,99	1 (1.1)	0 (0.0)	>0,99

An Adverse Event was considered as related with study medication if the relationship specified was: ‘Definite relationship’, ‘Likely relationship’ or ‘Possible relationship’.

The most frequently reported AEs were gastrointestinal disorders [Ibuprofen: 14 patients (13.08%); placebo: 8 patients (8.10%)], general disorders and administration site conditions [Ibuprofen: 10 (9.35%); placebo: 10 (10.10%)]. Data in Table B in S2 Table summarizes the most frequent AE; the difference between both treatment arms was not statistically significant.

In both treatment arms, pain and burning at the injection site were reported at some time point in more than half of the patients, while erythema and induration happened in few cases (Table C in S3 Table), no statistically significant differences were found.

## Discussion

Despite the wide range of currently available medications and techniques for pain management, we have not been able to ensure that patients will not have pain in the postoperative period [16,17] and consequently they will be exposed to the multiple complications that can result from improper treatment of pain [2,5,10,18,19]. The main objective of the study was to evaluate the efficacy of IV-Ibuprofen for the management of postoperative pain in comparison to placebo, while the secondary objective was to evaluate tolerability and safety of IV-Ibuprofen. The primary endpoint was reached, as intravenous treatment with ibuprofen reduced the morphine requirements within the 24 hours post-surgery, to almost half of the mean compared to placebo group. In comparison to previous studies conducted with intravenous formulations of ibuprofen [8,9,13] the median morphine consumption was lower in both treatment arms, which may have been due to the very different type of surgery conducted, possibly associated with lower levels of post-operative pain. For example in the study of Southwoth et al. 73% of patients underwent abdominal surgical procedures, fifty-five per cent of which were hysterectomies (predictive VAS 7 to 10). However, while the median morphine consumption was higher, the total morphine reduction was only 26%, compared to a 47.6% reduction in our study. A similar outcome were observed in both Singla et al. study, where patients went through orthopaedic surgery receiving iv-ibuprofen used 30.9% less morphine than those receiving placebo, and the Kroll et al. study, where the median morphine requirements was reduced by 19% in patients that experience abdominal hysterectomy receiving IV-Ibuprofen.

Furthermore related to morphine requirements, both the number of morphine doses and the number of attempts of dosing morphine at PCA were lower in patients treated with ibuprofen than in patients treated with placebo. Treatment with IV-Ibuprofen had an additional benefit for those patients because they had also a lower risk for treatment failures versus patients treated with placebo.

Since patients could self-administer morphine via a PCA in both treatment arms it was expected that pain would be well controlled in most of the patients. However, acute pain both at rest and at movement within 24 hours after surgery was even better controlled in patients treated with ibuprofen than in patients treated with placebo, with this difference being statistically significant. It has been reported that postoperative mean pain scores higher than 3 have a significant effect on general activity, mood, walking ability, and sleep [20]. These results suggest that adding IV-Ibuprofen could have a clinical benefit in postoperative pain control, especially during the first 24 hours [21]. Other secondary endpoints such as Ramsay-Hunt sedation scale and nocturnal awakenings due to pain did not show significant differences between both treatment groups.

The major weakness of our study concerns both the sample size and the projected morphine consumption, as we had planned to analyse patients from two distinct groups, orthopaedic and abdominal surgery. The literature describes orthopaedic surgery as one of the most painful surgeries (postoperative pain prediction of VAS score from 7 to 10). Not being able to include orthopaedic surgery patients had major implications on the outcome of the study: it lowered the total VAS score prediction of severe postoperative pain resulting in a lower range; it prevented us from being able to assess the effectiveness of the use of iv ibuprofen in orthopaedic surgery; and it precluded us from being able to draw conclusions about the use of IV-Ibuprofen in a longer period (72 hours), and the benefit of reduced morphine consumption during the three days post- surgery. Regarding the lower average consumption of morphine in our study, initially we predicted that a reduction of 10 mg of morphine consumption would be needed to be statistically significant. But since we could only evaluate the effectiveness of the abdominal surgery patients, which were predominantly laparoscopic surgery, a mean reduction of 7 mg or a median reduction of 15.66 mg in morphine consumption seems to be clinically relevant, regardless the result was statistically significant. Although our study was not designed to evaluate the clinical significance of the decrease in morphine requirements, current literature suggests the negative influence of morphine on the immune system, affecting cancer recurrence in a dose dependent way [22,23]. Besides the well-known classic opioids-related adverse effects on recovery and rehabilitation [3], another reasons for trying to diminish morphine consumption are on one hand that many patients have comorbidities and are poly-medicated which interfere with pharmacodynamics of the opioids and increase even more the risk of drugs interaction and side effects [24]; and on the other hand the probability of development of tolerance and hyperalgesia induced by opioids [25,26]. Hence, all opioid dose-sparing strategies are desired [27].

The safety analysis included all 206 patients that were treated in the study. Ibuprofen was safe and well tolerated, local injection site reactions and laboratory data did not reveal any additional risk related to intravenous infusion of ibuprofen. No substantial differences between the two treatments arms were note with regards to the incidence of AE. Some events such as urinary retention, headache, nervous system disorders and enzyme alterations (increased transaminases) were more frequent in the placebo-treated group, while nausea and vomiting were more frequent in the ibuprofen-treated group. It should be noted that nausea, vomiting and abdominal pain are common specific complications after abdominal surgery [28] (which comprised the majority of our patients). Therefore, not necessarily attributable to the drugs used, but it could be involved both genetic factors as well as the type of surgery [28,29]. All serious adverse events were unrelated to the study treatment and were resolved. There were no deaths during the study and no prolonged hospitalizations due to adverse events related to the study treatments. The incidence of treatment emergent adverse events, which constitute typical opioids adverse reactions, was very similar in both treatment arms, despite the significant reduction of morphine consumption in the ibuprofen arm versus the placebo arm. There were no reports of bronchospasm, constipation, dry mouth, muscle twitching, sleepiness, sweating, respiratory depression and confusion. The results from this trial agree with the results from other trials conducted in surgical patients treated with other approved IV-Ibuprofen formulations. In concordance with our results the trial from Southworth et al., Kroll et al., and Singla et al., also show that similar treatment emergent adverse events occurred in both study groups and there were no significant differences in the incidence of serious adverse events, neither gastrointestinal toxicity, bleeding or renal failure [30]. Moreover, with respects to the warnings of the European Medicines Agency (EMA) about the long term use and/or at high doses of Ibuprofen and other NSAID at the level of the renal, gastrointestinal, hematologic, bone, cardiovascular function among others, and considering the safety profile demonstrated both in our study and previous, the use of IV-Ibuprofen for a short period of time in in hospitalized patients should not be an issue.

Although our results from orthopaedic surgery patients were not available for an efficacy analysis, due to the small sample size, the safety and tolerability results suggest that IV-Ibuprofen may be also safe and well tolerated when infusions are administered throughout 48 or 72 hours after surgery, though further studies are required. This is the first European phase III clinical trial about the use of IV-Ibuprofen. It is a multicentre national study, with a double blind and randomized controlled design. Conducting a study in our local population is important since it is known that sex, age, weight, cultural factors as well as prior experiences of patients may impact pain response [31,32].

## Conclusions

Perioperative administration of IV-ibuprofen 800 mg every 6 hours in abdominal surgery patients decreases morphine requirements, pain, and the risk of rescue treatment with other analgesics. IV-ibuprofen was safe and well tolerated. Treatment with IV-ibuprofen 800 mg every 6 hours could be a useful adjuvant for short-term use in perioperative pain in abdominal surgery in adult patients. Given the safety profile of IV-ibuprofen, the save on morphine consumption and its known anti-inflammatory properties beneficial not only on postoperative pain relief, but on the pathophysiological mechanisms of acute postoperative pain, we consider appropriate that protocols for management of postoperative pain should include IV-ibuprofen 800 mg every 6 hours as an option to offer patients an analgesic benefit while reducing the potentially risks associated with morphine consumption.

## Supporting Information

S1 File Trial Protocol. (PDF)

S2 File CONSORT Checklist. (PDF)

S1 Table Serious Adverse Events. (PDF)

S2 Table Most frequent treatment emergent adverse events. (PDF)

S3 Table Summary of local reactions. (PDF)
