# Supplementary material for: Intravenous Ibuprofen for Treatment of Post-Operative Pain: A Multicenter, Double Blind, Placebo-Controlled, Randomized Clinical Trial
Source: PLoS One. 2016 May 6;11(5):e0154004. doi: 10.1371/journal.pone.0154004 (PMC4859493; doi:10.1371/journal.pone.0154004)
Supplement: S1 File — (PDF) [file pone.0154004.s001.pdf]

A MULTICENTER, RANDOMIZED, DOUBLE-BLIND, PLACEBO-CONTROLLED TRIAL TO EVALUATE EFFICACY AND SAFETY OF A NEW FORMULATION OF IBUPROFEN 800 MG EVERY 6 HOURS IN THE MANAGEMENT OF POSTOPERATIVE PAIN

STUDY CODE: BIBEC02

EUDRA-CT CODE: 2011-005007-33

STUDY SPONSOR: Laboratorios Biomendi S.A.U

Confidentiality Statement

The information in this document is confidential and is not to be disclosed without the written consent of Laboratorios Biomendi"

1. SIGNATURES PAGE

I am aware of, and agree to comply with, all of the procedures contained within the current protocol and informed consent form (ICF) listed below:

- Protocol: BIBEC02, version 1, 20th October 2011
- ICF: version 1, 20th October 2011

Study Coordinator
Dr. Francisco Abad Santos
Hospital Universitario de la Princesa

30/10/2011
Date / Signature

Study Coordinator
Concepción Pérez Hernández
Hospital Universitario de la Princesa

30/10/11
Date / Signature

Study Coordinator
Dolores Ochoa Mazarro
Hospital Universitario de la Princesa

30/10/11
Date / Signature

Study Coordinator
Antonio Planas
Hospital Universitario de la Princesa

30/10/2011
Date / Signature

Sponsor
D. Ignacio Ortúzar
Laboratorios Biomendi S.A.U.

30/10/2011
Date / Signature

2. SYNOPSIS

0. Type of application

Phase III efficacy and safety study

1. Sponsor

D. Ignacio Ortúzar

Laboratorios Biomendi S.A.U.

Pol. Ind. de Bernedo S/N

01118 Bernedo - Álava

Phone: +34 917 104 007 Fax: +34 917 103 630

e-mail: iortuzar@gesgenericos.com

2. Full title of the trial

A multicenter, randomized, double-blind, and parallel groups, placebo-controlled trial of the efficacy and safety of a new formulation of ibuprofen 800 mg every 6 hours in the management of postoperative pain.

3. Protocol Codes

STUDY CODE: BIBEC02

EUDRA CT number: 2011-005007-33

4. Study Coordinators and work address

Concepción Pérez Hernández, MD, PhD[1]

Francisco Abad Santos, MD, PhD[2]

Dolores Ochoa Mazarro, MD[2]

Antonio Planas, MD, PhD[3]

[1]Pain Unit

[2]Clinical Pharmacology Department

[3]Anesthesiology Department

Hospital Universitario de la Princesa

Diego de León 62

28006 MADRID, SPAIN.

Phone: +34 91 520 24 25.

Fax: +34 91 520 25 40

5. Study centres

The investigators and centres will be listed at **Clinical trial Application Form.**

Coordinating center: Hospital Universitario de la Princesa, Madrid. Dra. Concepción Pérez.

6. Reference Ethics Committee

The reference Ethics Committee will be the Ethics Committee for Clinical Investigation of Hospital Universitario de la Princesa. Approval by the Ethics Committees of the participating centres is required.

7. Study monitor

Pivotal S.L.

Calle Gobelas 19

28023 La Florida Madrid

Tel: +34 917 081 250

e-mail: miriam.lozano@pivotal.es

8. Treatment

Test drug: 800 mg: ibuprofen (INN) 800 mg/200 mL (4 mg/mL) intravenous solution manufactured by Laboratorios Biomendi S.AU. 800 mg will be administered in each scheduled dose as a 15 minutes infusion

Placebo: a saline infusion of the same volume will be administered to those patients randomized to the placebo arm.

Treatment will be administered every 6 hours until end of PCA (patient controlled analgesia) with a minimum of 24 h and a maximum of 72 h.

9. Clinical Trial Phase

Phase III

10. Objectives

Main objective: to study the efficacy of intravenous administration of ibuprofen compared to placebo in patients with postoperative pain.

Secondary objective: To evaluate its tolerability and safety profile.

11. Study design

This is a phase III, national, multicenter, randomized, double-blind, parallel groups, and placebo-controlled.

Patients will be randomized to receive either ibuprofen or placebo. Stratified randomization will be used to assign eligible patients to a stratum according to type of surgery and centre. Randomization sequence will be generated in the coordinating centre.

Blinding of the patient and study personnel evaluating response will be warranted by adequate drug labelling.

12. Disease or disorder under study

Moderate to severe postoperative pain.

13. Patient eligibility

Patients will be required to meet all the following **inclusion criteria**:

1. Men or women between 18 and 80 years old.
2. Being scheduled for elective single surgical site orthopaedic surgery (hip or knee joint replacement), or abdominal surgery (inguinal hernia, cholecystectomy)
3. Being scheduled for general anaesthesia.
4. Having anticipated need for postoperative narcotic analgesia administered by patient controlled analgesia (PCA).
5. Expected to stay at the hospital for at least 24 h.
6. Providing written informed consent for participating in this study.

Patients will be excluded from the study if they have any of the following e**xclusion criteria**:

1. Use of NSAID within 12 hours prior to the first planned dose.
2. Taking oral anticoagulants, lithium, combination of ACE inhibitors, furosemide or aspirin.
3. Anaemia (haemoglobin <10 g/dl) and/or history or evidence of asthma or heart failure.
4. History of allergy or hypersensitivity to any component of IV ibuprofen, aspirin or aspirin related products, NSAID or COX-2 inhibitors.
5. Pregnant or nursing.
6. Weight less than 40 kg.
7. History of severe head trauma that required hospitalization, intracranial surgery or stroke within the previous 30 days, or any history of intracerebral arteriovenous malformation, cerebral aneurism or CNS mass lesion.
8. History of congenital bleeding diathesis or any active clinically significant bleeding or underlying platelet dysfunction.
9. Gastrointestinal bleeding that required medical intervention.
10. Platelet count less than 80.000 determined within the 28 days prior to surgery.
11. Pre-existing dependence on narcotics or receiving chronic treatment with opioids.

12. Severe renal failure (calculated creatinine clearance < 60 ml/min).
13. Liver failure, ALAT or ASAT >3 times upper limit of normality, or billirubin >2 g/dl.
14. Diagnosed of Bowel Inflammatory Disease.
15. Not able to understand the requirements of the study, or to abide by the study restrictions or to return for the required assessments.

14. Response Assessment

The **main objective** of the study will be to evaluate the efficacy of intravenous ibuprofen for the management of postoperative pain in comparison to placebo. The primary efficacy endpoint will be the reduction in total morphine use in the first 24 hours post- surgery as compared to placebo.

The secondary efficacy endpoints will be the following:

- Consumption of morphine in the first 48 h (and 72 h) hours post- surgery.
- Pain intensity at rest and with movement measured with the eleven points visual analogue scale (VAS), at 1 and 3 hours and every 6 hours thereafter up to hour +24h, and then every 8 h up 6 hours after last ibuprofen dosing.
- Ramsay-Hunt sedation scale.
- Time to first subsequent narcotic analgesia (or time to treatment failure).
- Number of doses of morphine and number of attempts of dosing at PCA.
- Nocturnal awakenings due to pain.

The **secondary objective** will be to evaluate tolerability and safety of IV ibuprofen. Safety measurements will consist on the following:

- Report of adverse events (AEs) during the study
- Local reactions due to IV infusion (pain, erythema, phlebitis)
- Vital signs (heart rate, blood pressure, temperature) at 1, 3, 6 hour after the initial dose and every 8 hours thereafter.
- Routine laboratory tests (chemistry, haematology and coagulation) at baseline and within 24 hours after the last dose.

15. Statistical analysis and sample size calculation

The primary efficacy endpoint will be the consumption of morphine in the first 24 hours post-surgery. In the following table we can see the morphine requirements (mean and SD) during the immediate 24 hours following surgery in published studies with IV ibuprofen:

**Reference**	**Type of patients**	**Ibuprofen 800 mg /6 h**	**Placebo**
Southworth et al, 2009(1)	272 adult patients undergoing elective, single-site orthopaedic or abdominal surgery	43.8 (33.7)	48.9 (27.7)
Singla et al, 2010(2)	185 adult patients undergoing elective orthopaedic surgery	41.1 (27.3)	59.5 (29.9)
Kroll et al, 2011(3)	319 patients undergoing total abdominal hysterectomy surgery	47.3 (25.6)	55.9 (20.6)

If we consider that morphine requirement in the placebo group will be 55 mg and the ibuprofen treatment will reduce it to 45 mg, and accepting an alpha risk of 0.05 and a beta risk of 0.2 in a two-sided test, 143 subjects are necessary in each group to recognize as statistically significant a difference greater than or equal to 10 mg. The common standard deviation is assumed to be 30. Considering a drop out rate of 10-12%, a total of 320 patients will be included, 160 in each treatment group. If half of the patients corresponds to each type of surgery (orthopaedic surgery or abdominal surgery), we will have a power of 55% to evaluate differences in each group.

16. Duration of treatment

Each of the patients that fulfil all the inclusion criteria and none of the exclusion criteria will be randomized to one of the following treatment groups:

<u>Active treatment group</u>: 800 mg IV ibuprofen, starting at the moment of skin closure and every 6 hours. Medication will be infused over 15 minutes.

<u>Placebo group</u>: 200 ml of saline solution, starting at the moment of after skin closure and every 6 hours. Medication will be infused over 15 minutes.

All patients will receive morphine administered by patient controlled analgesia (PCA). Investigation treatment will be administered every 6 hours until 24 h in abdominal surgery, 48 h in hip surgery and 72 h in knee surgery.

Patients will be followed up 3 ± 1 days after last ibuprofen dose to register any adverse event. If patient has been discharged from the hospital, this visit can be performed by phone.

17. Schedule and programmed date

The overall duration of the study is estimated at 1 year. Recruitment will be competitive among participating centres and will close 6 months after the first patient inclusion.

3. RESUMEN

0. Tipo de solicitud

Estudio de eficacia y seguridad de fase III

1. Promotor del ensayo

D. Ignacio Ortúzar

Laboratorios Biomendi S.A.U.

Pol. Ind. de Bernedo S/N

01118 Bernedo - Álava

Phone: +34 917 104 007 Fax: +34 917 103 630

e-mail: iortuzar@gesgenericos.com

2. Título completo del ensayo

Estudio multicéntrico, aleatorizado, doble ciego, de grupos paralelos, controlado con placebo, para evaluar la eficacia y seguridad de una nueva formulación de ibuprofeno 800 mg cada 6 horas para el manejo del dolor postoperatorio.

3. Códigos del protocolo

CÓDIGO DEL ESTUDIO: BIBEC02

Número EUDRA CT: 2011-005007-33

4. Coordinadores del estudio

Concepción Pérez Hernández, MD, PhD[1]

Francisco Abad Santos, MD, PhD[2]

Dolores Ochoa Mazarro, MD[2]

Antonio Planas, MD, PhD[3]

[1]Unidad del Dolor

[2]Servicio de Farmacología Clínica

[3] Servicio de Anestesiología

Hospital Universitario de la Princesa

Diego de León 62

28006 MADRID, SPAIN.

Phone: +34 91 520 24 25.

Fax: +34 91 520 25 40

5. Investigadores y centros colaboradores

Los centros e investigadores serán los incluidos en el formulario de solicitud.

Centro coordinador: Hospital Universitario de la Princesa, Madrid. Dra. Concepción Pérez.

6. Comité Ético de Investigación Clínica de Referencia

El Comité Ético de Investigación Clínica de Referencia será el del Hospital Universitario de la Princesa. El estudio además será presentado a los comités del resto de los centros participantes para su aprobación.

7. Monitorización del estudio

Pivotal S.L.

Calle Gobelas 19

28023 La Florida Madrid

Tel: +34 917 081 250

e-mail: miriam.lozano@pivotal.es

8. Tratamiento

Fármaco en estudio: ibuprofeno (INN) 800 mg/200 mL (4 mg/mL) solución para infusión intravenosa, fabricado por Laboratorios Biomendi S.AU. Se administrarán 800 mg cada 6 horas en una infusión de 15 minutos de duración.

Placebo: se administrará el mismo volumen de suero salino fisiológico, cada 6 horas, y el tiempo de infusión también serán 15 minutos.

El tratamiento se administrará cada 6 h hasta retirada de PCA con un mínimo de 24 h y un máximo de 72 h.

9. Fase del ensayo clínico

Fase III

10. Objetivos

Objetivo Principal: estudiar la eficacia de la administración de ibuprofeno intravenoso comparada con placebo en pacientes con dolor postoperatorio.

Objetivo secundario: evaluar su seguridad y tolerabilidad.

11. Diseño del estudio

Se trata de un estudio de fase III, nacional, multicéntrico, aleatorizado, doble ciego, de grupos paralelos, controlado con placebo.

Los pacientes que participen en el estudio serán asignados de forma aleatoria a recibir ibuprofeno o placebo. Se llevará a cabo una aleatorización estratificada por centro y tipo de cirugía. La secuencia de aleatorización se generará en el centro coordinador del estudio.

El enmascaramiento del paciente y el personal que evalúe la respuesta al tratamiento se garantizará con un adecuado etiquetado de la medicación del estudio.

12. Patología en estudio

Dolor postoperatorio de intensidad moderada o grave.

13. Selección de pacientes

Para ser incluidos en el estudio, los pacientes deberán cumplir todos los siguientes **criterios de inclusión**:

1. Hombre o mujer de 18 a 80 años.

2. Haber sido programados para cirugía ortopédica (prótesis de cadera o rodilla) o abdominal (herniorrafia inguinal o colecistectomía)
3. Que vaya a ser intervenido con anestesia general.
4. Que se prevea que van a precisar analgesia postoperatoria con opioides administrado por el propio paciente (PCA o analgesia controlada por el paciente)
5. Que vayan a permanecer en el hospital durante las 24 h siguientes a la cirugía.
6. Que otorgue el consentimiento informado por escrito.

Los pacientes no serán incluidos en el estudio si presentan alguno de los siguientes **criterios de exclusión**:

1. Uso de antiinflamatorios no esteroideos (AINE) en las 12 h previas a la administración de la primera dosis del estudio.
2. En tratamiento con anticoagulantes orales, litio, combinaciones de furosemida e inhibidores de la enzima convertidora de angiotensina, o aspirina.
3. Anemia (hemoglobina <10 g/dl) y/o antecedentes o evidencia actual de asma o insuficiencia cardíaca.
4. Antecedentes de alergia o hipersensibilidad a cualquiera de los componentes de la formulación de ibuprofeno intravenoso, aspirina o productos relacionados, otros AINE (incluyendo inhibidores de la COX-2).
5. Embarazo o lactancia.
6. Peso menor de 40 kg.
7. Antecedentes de traumatismo craneoencefálico severo con ingreso hospitalario, cirugía intracranial o ictus en los 30 días previos al inicio del estudio, así como antecedentes de malformación arteriovenosa, aneurismas o lesiones ocupantes de espacio a nivel cerebral.
8. Antecedentes de diátesis hemorrágica congénita o cualquier hemorragia activa clínicamente relevante, así como antecedentes de disfunción plaquetaria subyacente.
9. Antecedentes de hemorragia gastrointestinal que haya precisado intervención médica.
10. Recuento plaquetario menor de 80.000 dentro de los 28 días previos a la cirugía.

11. Dependencia de narcóticos o tratamiento crónico con opioides.
12. Insuficiencia renal (aclaramiento de creatinina calculado <60 ml/min).
13. Insuficiencia hepática, ALAT o ASAT >3 veces el límite superior de la normalidad o bilirrubina >2 g/dl.
14. Diagnostico de enfermedad inflamatoria intestinal
15. Sin capacidad para comprender los requerimientos del estudio, o que no esté dispuesto a acatar las restricciones del estudio o a acudir a las visitas necesarias.

14. Evaluación de la respuesta

El objetivo principal del estudio es evaluar la eficacia de la administración de ibuprofeno intravenoso comparado con placebo en el manejo del dolor postoperatorio. La variable primaria de eficacia será la reducción en el uso de morfina en las primeras 24 h en comparación con placebo.

Las variables secundarias de eficacia serán:

- Reducción en el consumo de morfina a las 48 h (y 72) postcirugía.
- Intensidad del dolor en reposo y con movimiento medido a través de la Escala Visual Analógica (EVA), 1 y 3 h tras la primera dosis, cada 6 horas hasta las 24 h y después cada 8 h hasta 6 h después de la última dosis de ibuprofeno.
- Escala de sedación de Ramsay-Hunt.
- Tiempo hasta la administración de la primera dosis de analgesia de rescate (o tiempo hasta el fracaso del tratamiento).
- Número de dosis de morfina y número de intentos de dosis de morfina en PCA.
- Despertares nocturnos por dolor.

El **objetivo secundario** del estudio es evaluar la tolerabilidad y seguridad de la administración de ibuprofeno intravenoso. Las variables a recoger serán las siguientes:

- Registro de Efectos Adversos (EA) durante el estudio.
- Reacciones locales en el lugar de inyección (dolor, eritema, flebitis)

~ Constantes vitales (frecuencia cardíaca, frecuencia respiratoria, tensión arterial, temperatura) 1, 3 y 6 horas tras la dosis inicial y posteriormente cada 8 h.

~ Analíticas de rutina que incluirán hematología, bioquímica y coagulación, y se llevarán a cabo antes del inicio del tratamiento y en las 24 horas después d ela última dosis de ibuprofeno.

15. Análisis estadístico y cálculo del tamaño muestral

La variable primaria de eficacia es la reducción en el consumo de morfina en las primeras 24 horas postcirugía. En la siguiente tabla se recogen los requerimientos medios de morfina en el grupo de ibuprofeno intravenoso y placebo durante las primeras 24 h tras la cirugía publicados en estudios de eficacia de ibuprofeno intravenoso:

**Referencia**	**Tipo de pacientes**	**Ibuprofeno 800 mg /6 h**	**Placebo**
Southworth et al, 2009(1)	272 pacientes postcirugía ortopédica o abdominal	43.8 (33.7)	48.9 (27.7)
Singla et al, 2010(2)	185 pacientes postcirugía ortopédica	41.1 (27.3)	59.5 (29.9)
Kroll et al, 2011(3)	319 pacientes posthisterectomía	47.3 (25.6)	55.9 (20.6)

Si consideramos que los requerimientos de morfina en el grupo de placebo será de 55 mg y en el grupo de ibuprofeno de 45 mg, aceptando un error alfa de 0.05 y beta de 0,2 en un test bilateral, se necesitarán 143 pacientes para detectar una diferencia estadísticamente significativa mayor o igual a 10 mg. Se asume una desviación estándar común de 30. Contando con una tasa de abandonos del 10-12%, se necesitarán un total de 320 pacientes, 160 en cada grupo de tratamiento. Si la mitad de los pacientes corresponden a cada tipo de cirugía (ortopédica o abdominal) tendremos un poder del 55% para evaluar las diferencias.

16. Duración del tratamiento

Los pacientes que cumplan todos los criterios de inclusión y ninguno de los de exclusión serán aleatorizados a uno de los siguientes grupos de tratamiento:

Grupo de tratamiento activo: 800 mg de ibuprofeno intravenoso, iniciado durante la sutura de la herida quirúrgica y cada 6 horas. La medicación se infundirá en 15 minutos.

Grupo Placebo: 200 ml de suero salino fisiológico, iniciado durante la sutura de la herida quirúrgica y cada 6 horas. La medicación se infundirá en 15 minutos.

Todos los pacientes recibirán morfina administrada según necesidades (PCA o patient controlled analgesia). El tratamiento en investigación se administrará cada 6 horas hasta 24 h en cirugía abdominal, 48 h en cirugía de cadera y 72 h en cirugía de rodilla.

Se realizará una visita de seguimiento a los 3 ± 1 días de la última dosis de ibuprofeno para registrar la aparición de efectos adversos. Si el paciente ha recibido el alta hospitalaria, esta visita se puede realizar por teléfono.

17. Calendario y fechas previstas

La duración total del estudio se estima en 1 año desde la inclusión del primer paciente. El reclutamiento será competitivo entre centros y se cerrará a los 6 meses de inclusión del primer paciente.

4. CONTENTS

1. SIGNATURES PAGE 2
2. SYNOPSIS 3
3. RESUMEN 10
4. CONTENTS 17
5. GENERAL INFORMATION 19
5.1. IDENTIFICATION OF THE TRIAL 19
5.2. NAME AND ADRESS OF THE SPONSOR 19
5.3. NAME AND ADRESS OF THE MONITOR 19
5.4. STUDY COORDINATORS 19
5.5. PARTICIPATING CENTRES CONFIRMAR 20
5.6. TRIAL DESIGN 20
5.7. TREATMENT DESCRIPTION 20
5.8. STUDY SCHEDULE 20
6. INTRODUCTION 21
7. STUDY OBJECTIVES AND VARIABLES 25
7.1. MAIN OBJECTIVE 25
7.2. SECONDARY OBJECTIVE 25
8. PATIENT ELIGIBILITY 26
8.1. INCLUSION CRITERIA 26
8.2. EXCLUSION CRITERIA 26
9. STUDY DESIGN AND DEVELOPMENT 27
9.1. TREATMENT DESCRIPTION 27
9.2. RANDOMIZATION PROCESS 28
9.3. BLINDING PROCEDURE 28
9.4. FOLLOW-UP AND DISCONTINUATION CRITERIA 28
9.5. STUDY PROCEDURES AND RESPONSE ASSESMENT 29
10. STATISTICAL ANALYSIS 31
10.1. SAMPLE SIZE CALCULATION 31
10.2. STATISTICAL ANALYSIS 31
11. SAFETY ASSESSMENT 32
11.1. MINIMUM INFORMATION TO BE SPECIFIED 32
11.2. IMPUTABILITY CRITERIA 33
11.3. INTENSITY CLASSIFICATION 33
11.4. SERIOUS AND UNEXPECTED ADVERSE EVENTS 33
11.5. SERIOUS ADVERSE EVENT REPORTING PROCEDURES 34
11.6. GENERAL AND SPECIFIC RULES FOR THE INVESTIGATORS 34
12. ETHICS 35
13. DATA HANDLING AND RECORD KEEPING 36
13.1. DOCUMENTATION ARCHIVE 36
13.2. MEDICATION MANAGEMENT 36

14. FINANCING AND INSURANCE 36
14.1. FINANCING 36
14.2. INSURANCE 36
15. CONFIDENTIALITY AND DATA PROTECTION 37
16. PUBLICATION POLICY 37
17. REFERENCES 38
18. APPENDIX 40
18.1. DECLARATION OF HELSINKI 40
18.2. SERIOUS OR UNEXPECTED ADVERSE EVENTS SPANISH REPORT FORM 44
18.3. PATIENT INFORMATION SHEET AND INFORMED CONSENT FORM 46
18.4. CASE REPORT FORM 52
18.5. INVESTIGATOR BROCHURE 53

5. GENERAL INFORMATION

5.1. Identification of the trial

STUDY CODE: BIBEC02

EUDRA CT number: 2011-005007-33

Title: Multicenter, randomized, double-blind, and parallel groups, placebo-controlled trial of the efficacy and safety of a new formulation of ibuprofen 800 mg every 6 hours in the management of postoperative pain.

5.2. Name and Address of the Sponsor

D. Ignacio Ortúzar
Laboratorios Biomendi S.A.U.
Pol. Ind. de Bernedo S/N
01118 Bernedo - Álava
Phone: +34 917 104 007 Fax: +34 917 103 630
e-mail: iortuzar@gesgenericos.com

5.3. Name and address of the monitor

Pivotal S.L.

Calle Gobelas 19

28023 La Florida Madrid

Tel: +34 917 081 250

e-mail: miriam.lozano@pivotal.es

5.4. Study Coordinators

Concepción Pérez Hernández, MD, PhD[1]
Francisco Abad Santos, MD, PhD[2]
Dolores Ochoa Mazarro, MD[2]
Antonio Planas, MD, PhD[3]
[1]Pain Unit
[2]Clinical Pharmacology Department
[3]Anesthesiology Department
Hospital Universitario de la Princesa
Diego de León 62

28006 MADRID, SPAIN.
Phone: +34 91 520 24 25.
Fax: +34 91 520 25 40

5.5. Participating centers

The investigators and centres will be listed at **Clinical trial Application Form.**

Coordinating center: Hospital Universitario de la Princesa, Madrid. Dra. Concepción Pérez.

5.6. Trial design

This is a phase III, national, multicentre, randomized, double-blind, placebo controlled, parallel groups study.

5.7. Treatment description

Patients included in the study will be randomly assigned to one of the following groups:

Treatment Group: patients will receive ibuprofen (INN) 800 mg/200 mL (4 mg/mL) intravenous solution manufactured by Laboratorios Biomendi S.AU. 800 mg will be administered in each scheduled dose as a 15 minutes infusion

Placebo Group: a saline infusion of the same volume will be administered to those patients randomized to the placebo arm.

5.8. Study schedule

The overall duration of the study is estimated at 1 year. Recruitment will be competitive among participating centres and will close 6 months after the first patient inclusion.

6. INTRODUCTION

Acute pain is a significant problem for inpatients and can occur secondary to acute illness or disease processes, trauma, or operative procedures. In the post-operative period, 80% of individuals suffer from post-operative pain with almost all describing it as moderate to severe (4). Physiologically, pain serves to alert individuals to tissue damage and to prevent further harm. However, in the acute inpatient setting, it often serves no useful purpose. Pain activates the sympathetic nervous system, increasing blood pressure, cardiac workload and respiratory rate (5). It impairs recovery by reducing mobility and physical activity. Despite the use of medications to control pain, it often remains undertreated and is a problem in hospitals and long-term care facilities.

Opioid analgesics are a mainstay in the management post-operative and acute pain in the inpatient setting (1). However, their use is often limited by adverse effects including respiratory depression, sedation, allergic reactions, and gastrointestinal events. Opioids are useful in mitigating the sensation of pain, but provide no benefits to the underlying disease process. Adjunctive agents for pain including non-steroidal anti-inflammatory agents (NSAIDs) may be used in combination with opioids. Combination therapy may help mitigate the side effects of both agents by reducing the total dose required. The anti-inflammatory properties of NSAIDs may also be useful in promoting healing and resolution of pain. NSAIDs inhibit the production of cyclooxygenase (COX)-1 and COX-2 enzymes and prevent the sensitization of pain receptors at the site of injury. The World Health Organization (WHO) includes NSAIDs in step 1 of the analgesic ladder for the treatment of mild to moderate pain (6). They are also included as adjuncts in the treatment of persistent and moderate to severe pain in steps 2 and 3, respectively.

The antipyretic effects of NSAIDs are also well documented, resulting from inhibition of COX-1 to block the conversion of arachidonic acid to prostaglandins such as PGE2 in the immune response pathway (7). Fever is common in hospitalized patients and can be a result of infection, thrombosis, autoimmune disorders, malignancies, drugs, or other unknown causes (8). Reduction of fever not only offers substantial benefit to a patient’s well-being but the metabolic compromise of sustained fever may potentiate risks associated with common co-morbidities encountered in hospitalized patients.

There are a few NSAID available in intravenous form in Spain. Desketoprofen (Enantyum®, Menarini Laboratories) and Ketorolac (Toradol®, Roche Pharma Laboratories),

although with known antipyretic effects, are most frequently used in the management of acute and post-operative pain. Their use is limited mainly by their side effects, and treatment for more than 2 days is not recommended (9). Many practitioners also have the perception that they are more effective than oral ibuprofen (10). However, a review of studies examining both parenteral ketorolac and oral ibuprofen found that oral ibuprofen is as effective as ketorolac in the management of pain in emergency department patients. The authors of this study also point out that the cost of oral ibuprofen is significantly less than parenteral ketorolac. Additional intravenous options available in Spain are lysine acetylsalicylate (Inyesprin®, Grunenthal Laboratories), Indometacin (Inacid DAP®, Lundbeck Pharm Laboratories) and Parecoxib (Dynastat®, Pfizer Laboratories). Parecoxib is the first COX-2 inhibitor available for parenteral administration and is approved through much of Europe for short-term perioperative pain control (11).

Oral ibuprofen is a commonly used NSAID with antipyretic, anti-inflammatory, and analgesic properties. It is a mainstay in the treatment of acute pain and fever and is readily available over the counter. Mechanistically, it acts both centrally and peripherally to reduce pain and fever. Ibuprofen is a racemic mixture of [-]R- and [+]S-isomers (12). The [+]S-isomer is responsible for clinical activity according to in vivo and in vitro studies. The [-]R-isomer is slowly and incompletely interconverted into the active [+]S-species in adults. Active drug levels are maintained by circulating reservoir of the [-]R-isomer. As described by early models of the oral dosage form, ibuprofen best fits a linear or first order pharmacokinetic compartment model. Receipt of 5 to 10 mg/kg of oral ibuprofen suspension or solution results in a time to achieve maximum concentration (tmax) of 0.75 to 1.5 hours (13)(14)(15). Elimination half-life is approximately 1.6 hours. The AUC of plasma ibuprofen is dose-dependent due to the concentration-dependent protein binding where doubling of the dose (5 to 10 mg/kg) results in a 1.6-fold increase in drug exposure (14). Ibuprofen is highly protein bound (99%). The protein binding is saturable and at concentrations .20 µg/mL becomes nonlinear. The estimated volume of distribution is 0.11 to 0.21 L/kg based on data from oral ibuprofen and there is a known age- and fever-related change (14)(15)(16)(17). The terminal half-life of intravenous ibuprofen appears to be slightly above 2 hours (mean = 2.26 hours) and consistent across all dosing ranges (12). Similar to data from the oral dosage form, antipyretic effects are delayed in relation to peak concentrations and last longer despite declining plasma ibuprofen concentrations. A summary of the pharmacokinetic parameters of intravenous ibuprofen from initial studies are detailed in Table 1.

Table 1 Summary of pharmacokinetic parameters of intravenous ibuprofen

Parameter	100 mg dose	200 mg dose	400 mg dose	800 mg dose
**Study:** Morris et al[33]				
Number of patients	31	30	31	–
AUC (µg·h/mL)	22.33 (12.75)	32.62 (17.39)	70.64 (31.93)	–
C_{max} (µg/mL)	12.17 (6.78)	18.93 (10.50)	39.76 (17.75)	–
$T_{1/2}$ (h)	2.47 (1.15)	2.11 (1.05)	2.26 (0.95)	–
**Study:** Intravenous ibuprofen product labeling; Data on file with Cumberland Pharmaceuticals®[25,34]				
Number of patients	–	–	12	12
AUC (µg·h/mL)	–	–	109.3 (26.4)	192.8 (18.5)
C_{max} (µg/mL)	–	–	39.2 (15.5)	72.6 (13.2)
$T_{1/2}$ (h)	–	–	2.22 (20.1)	2.44 (12.9)
**Study:** Pavliv et al[34]				
Number of patients	–	–	–	12
AUC (µg·h/mL)	–	–	–	195.7 (37.3)
C_{max} (µg/mL)	–	–	–	120.3 (13.5)
$T_{1/2}$ (h)	–	–	–	2.0 (0.5)

Important to note are the differences in infusion time among the three studies summarized (5- to 7-minute rapid infusion, 30-minute infusion, and 60-minute infusion) (12)(18)(19). Infusion time greatly influences maximum concentration (Cmax) and tmax, which is typically immediately following the end of the infusion. In a pharmacokinetic study of 24 healthy volunteers, dose of 400 mg and 800 mg were administered over 60-minute infusions (12). The association between the different doses demonstrates a Cmax ratio of 1.7 and an AUC ratio of 1.8 for the 400 mg and 800 mg doses, respectively. These data are similar to the pharmacokinetics observed with oral ibuprofen. Unpublished data of a randomized, cross-over, placebo controlled study, examined the pharmacokinetic profile of an 800-mg dose given via rapid infusion over 5 to 7 minutes (19). Twelve healthy volunteers, mean age of 31.7 years and mean weight of 77 kg, were enrolled. At approximately three hours post-dose, the mean serum concentration was 20 µg/mL with a half-life of approximately 2 hours. The AUCt-inf of 196 µg·h/mL (±37) is similar to previous pharmacokinetic studies in subjects receiving 800 mg. Compared to oral dosing of 800 mg in the same population, the AUCs were very similar (196.4 vs 195.7), but Cmax nearly doubled (120.3 vs 62.8) with significantly shorter time to peak concentrations (6.5 vs 90 minutes).

The use of ibuprofen in the inpatient or post-operative setting has previously been limited by the lack of a commercially available parenteral formulation. The drug's lipophilic properties have rendered production into an intravenous formulation difficult. Ibuprofen is available in an intravenous solution (Pedea®) only for treatment of patent ductus arteriosus in

the neonatal population (20). An intravenous form of ibuprofen (Caldolor®; Cumberland Pharmaceuticals, Nashville, TN, USA)(12) is already available in the United States. Phase III clinical efficacy studies performed in its clinical development program found that intravenous ibuprofen use improved pain control and reduced opioid use in the orthopedic and abdominal surgery setting as well as on fever reduction in the critically ill population(2)(1)(3).

BIOMENDI Laboratories is developing an intravenous formulation of ibuprofen for its approval in Spain. The aim of this study is to evaluate the efficacy and tolerability profile of IV ibuprofen in the management of acute moderate to severe post-operative pain.

7. STUDY OBJECTIVES AND VARIABLES

7.1. Main objective

The **main objective** of the study will be to evaluate the efficacy of intravenous ibuprofen for the management of postoperative pain in comparison to placebo. The primary efficacy endpoint will be the reduction in total morphine use in the first 24 hours post- surgery as compared to placebo.

The secondary efficacy endpoints will be the following:

- Consumption of morphine in the first 48 h (and 72) hours post- surgery.
- Pain intensity at rest and with movement measured with the eleven points visual analogue scale (VAS), at 1 and 3 hours and every 6 hours thereafter up to hour 24 h, and then every 8 h up to 6 hour after the last ibuprofen dose.
- Ramsay-Hunt sedation scale.
- Time to first subsequent narcotic analgesia (or time to treatment failure).
- Number of doses of morphine and number of attempts of dosing at PCA

7.2. Secondary objective

The **secondary objective** will be to evaluate tolerability and safety of IV ibuprofen. Safety measurements will consist on the following:

- Report of adverse events (AEs) during the study
- Local reactions due to IV infusion (pain, erythema, phlebitis)
- Vital signs (heart rate, blood pressure, temperature) at 1, 3, 6 hour after the initial dose and every 8 hours thereafter.
- Routine laboratory tests (chemistry, haematology and coagulation) at baseline and within 24 hours after the last dose.

8. PATIENT ELIGIBILITY

8.1. Inclusion Criteria

Patients will be required to meet all the following **inclusion criteria**:

1. Men or women between 18 and 80 years old.
2. Being scheduled for elective single surgical site orthopaedic surgery (hip or knee joint replacement), or abdominal surgery (inguinal hernia, cholecystectomy)
3. Being scheduled for general anaesthesia.
4. Having anticipated need for postoperative narcotic analgesia administered by patient controlled analgesia (PCA).
5. Expected to stay at the hospital for at least 24 h.
6. Providing written informed consent for participating in this study.

8.2. Exclusion criteria

Patients will be excluded from the study if they have any of the following e**xclusion criteria**:

1. Use of NSAID within 12 hours prior to the first planned dose.
2. Taking oral anticoagulants, lithium, combination of ACE inhibitors, furosemide or aspirin.
3. Anaemia (haemoglobin <10 g/dl) and/or history or evidence of asthma or heart failure.
4. History of allergy or hypersensitivity to any component of IV ibuprofen, aspirin or aspirin related products, NSAID or COX-2 inhibitors.
5. Pregnant or nursing.
6. Weight less than 40 kg.
7. History of severe head trauma that required hospitalization, intracranial surgery or stroke within the previous 30 days, or any history of intracerebral arteriovenous malformation, cerebral aneurism or CNS mass lesion.

8. History of congenital bleeding diathesis or any active clinically significant bleeding or underlying platelet dysfunction.
9. Gastrointestinal bleeding that required medical intervention.
10. Platelet count less than 80.000 determined within the 28 days prior to surgery.
11. Pre-existing dependence on narcotics or receiving chronic treatment with opioids.
12. Severe renal failure (calculated creatinine clearance < 60 ml/min).
13. Liver failure, ALAT or ASAT >3 times upper limit of normality, or billirubin >2 g/dl.
14. Diagnosed of Bowel Inflammatory Disease.
15. Not able to understand the requirements of the study, or to abide by the study restrictions or to return for the required assessments.

9. STUDY DESIGN AND DEVELOPMENT

9.1. Treatment description

Patients that fulfil all the inclusion and none of the exclusion criteria and sign the informed consent form will be randomly assigned to receive either intravenous ibuprofen or placebo:

Treatment group: 800 mg IV ibuprofen, starting at the moment of skin closure and every 6 hours, infused over 15 minutes

Placebo group: 200 ml of saline solution, starting at the moment of skin closure and every 6 hours, infused over 15 min.

All patients will receive morphine administered by patient controlled analgesia (PCA). Investigation treatment will be administered every 6 hours until 24 h in abdominal surgery, 48 h in hip surgery and 72 h in knee surgery.

9.2. Concomitant treatment

All patients will receive morphine administered by patient controlled analgesia (PCA) following a similar schedule in all centres: 1 mg bolus dose with a close time of 5 min and a maximum of 10 mg in an hour and a maximum of 30 mg in 4 hours.

The patient can not received other analgesic treatments.

It is allowed the administration of other treatments such as antiemetic or gastric protectors, according to the usual practice in each centre. All drugs administered to each patient should be registered in the case report form.

9.3. Randomization process

Stratified randomization will be used to assign eligible patients to a stratum according to:

- Type of surgery (abdominal or orthopaedic)
- Participating centre.

Randomization sequence will be generated in the coordinating centre. In order to ensure balanced group sizes, blocked randomization will be performed.

To ensure allocation concealment, a central informatic procedure will be used.

9.4. Blinding procedure

Blinding of the patient and study personnel evaluating response will be warranted by adequate drug labeling.

9.5. Follow-up and discontinuation criteria

Patients will be followed up 3 ± 1 days after last ibuprofen dose to register any adverse event. If patient has been discharged from the hospital, this visit can be performed by phone.

Treatment will be discontinued if any of the following occurs:

- The patient receives either narcotic pain medication (other than morphine) or non narcotic pain medication (including another NSAID).
- The patient is able to tolerate oral pain medication.
- Pain resolves.
- There's a loss of intravenous access.
- The patient is discharged from the hospital before 48 hours post-surgery.

9.6. Study procedures and response assessment

Recruitment will be made among patients scheduled for elective orthopaedic (hip or knee joint replacement) or abdominal surgery (inguinal hernia, appendectomy), and will take place within the previous 2 weeks. Patients that fulfil all inclusion criteria and none of the exclusion criteria will be proposed to participate in the study. One of the investigators will explain the trial purpose, and the risk and benefits of participating. If the patient wants to be included in the study, he/she will have to sign an Informed Consent form. Data from patients willing to participate but not fulfilling the selection criteria will be recorded.

Those patients included in the study will be randomly assigned to one of the following treatment groups:

Treatment group: 800 mg IV ibuprofen, starting at the moment of skin closure and every 6 hours up to 72 hours after the first dose.

Placebo group: 200 ml of saline solution, starting at the moment of skin closure and every 6 hours up to 72 hours after the first dose.

Information regarding medical history, physical examination and analytical parameters will also be recorded in the Case Report Form (CRF). Data related to treatment administration together with efficacy and safety assessments will also be recorded in CRF

Study procedures are summarized in Table 1.

Table 1. Study Procedures

	SCREENING PERIOD	DAY 1							DAY 2			DAY 3			DAY 4-7
Time since treatment initiation	-14 to -1	0	+1h	+3h	+6h	+12h	+18h	+24h	+32h	+40h	+48h	+56h	+64h	+72h	followup
Inclusion/Exclusion Criteria	X														
Clinical History	X														
Concomitant Medication	X							X			X			X	
Physical examination	X							X			X			X	
Vital Signs [1]	X		X	X	X	X	X	X	X	X	X	X	X	X	
Blood Test [2]	X										X			X	X
Randomization		X													
Treatment Start		X													
Morphine usage (mg)								X			X			X	
Pain intensity (VAS)		X	X	X	X	X	X	X	X	X	X	X	X	X	
Ramsay-Hunt scale		X	X	X	X	X	X	X	X	X	X	X	X	X	
Nocturnal Awakenings due to pain								X			X			X	
Adverse events	X		X	X	X	X	X	X	X	X	X	X	X	X	X
1) Including temperature, heart rate, respiratory rate and supine blood pressure. 2) Within 24 h after las dose, including haematology, biochemistry, urinanalysis, urine pregnancy test and coagulation.															

10. Statistical Analysis

10.1. Sample size calculation

The primary efficacy endpoint will be the consumption of morphine in the first 24 hours post-surgery. In the following table we can see the morphine requirements (mean and SD) during the immediate 24 hours following surgery in published studies with IV ibuprofen:

**Reference**	**Type of patients**	**Ibuprofen 800 mg /6 h**	**Placebo**
Southworth et al, 2009 (1)	272 adult patients undergoing elective, single-site orthopaedic or abdominal surgery	43.8 (33.7)	48.9 (27.7)
Singla et al, 2010 (2)	185 adult patients undergoing elective orthopaedic surgery	41.1 (27.3)	59.5 (29.9)
Kroll et al, 2011 (3)	319 patients undergoing total abdominal hysterectomy surgery	47.3 (25.6)	55.9 (20.6)

If we consider that morphine requirement in the placebo group will be 55 mg and the ibuprofen treatment will reduce it to 45 mg, and accepting an alpha risk of 0.05 and a beta risk of 0.2 in a two-sided test, 143 subjects are necessary in each group to recognize as statistically significant a difference greater than or equal to 10 mg. The common standard deviation is assumed to be 30. Considering a drop out rate of 10-12%, a total of 320 patients will be included, 160 in each treatment group. If half of the patients corresponds to each type of surgery (orthopaedic surgery or abdominal surgery), we will have a power of 55% to evaluate differences in each group.

10.2. Statistical analysis

Statistical analysis will be performed by Biomendi Laboratories. For analysis purposes, three study populations are prospectively defined:

- Efficacy analysis will be based in the Intention To Treat (ITT) population, composed by all subjects that have been randomized.

- A second efficacy analysis will be performed over the Per Protocol (PP) population, composed by all subjects that have received the study medication for at least 24 hours

- Safety analysis will be based in the Safety Population, composed by all the ubjects that have received at least 1 dose of the study medication.

A descriptive analysis will be performed for variables recorded during the study. Quantitative data will be described as Mean and Standard Deviation (SD). Qualitative data will be described as frequencies or percentages with 95% confidence intervals. Differences between groups will be analysed through the T Student test for quantitative variables and the chi-squared test for qualitative ones. A difference will be considered significant if $p < 0.05$. All statistical analysis will be performed using SPSS 14.0 software (SPSS Inc., Chicago, IL, USA).

The primary efficacy analysis will be the reduction in total morphine requirements in the first 24 hours post-surgery as compared to placebo, expressed as mean reduction). The plan for efficacy and safety analysis includes two sided T test (or ANOVA) for continuous variables, Life-table and Cox´s Proportional Hazard Model for time to events and Chi-square for categorical variables.

11. Safety Assessment

During the study all adverse events occurring to patients will be recorded, regardless of the relationship to study medication.

An adverse event (AE) is defined as an undesirable medical occurrence in a patient or clinical research subject who has been administered a pharmaceutical or nutritional product, which is not necessarily related to this treatment. An AE can be a sign, symptom or abnormal laboratory finding or test result. The period for reporting an AE starts when the study drug is administered and ends with the last follow-up.

11.1. Minimum information to be specified

The team of investigators shall monitor the possible adverse events which could arise during the study, recording the time of appearance, duration, intensity, evolution and

outcome, in order to evaluate the causal relationship between the adverse event and the medication.

Adverse events are gathered by asking a generic question like "Have you noticed anything since you took the medication?" or by spontaneous notification by a patient.

11.2. Imputability criteria

Adverse events will be classified according to the following criteria (14):

1. **Probable**: the AE can be classified as probably related to the medication if its timing is clearly related to the administration of the study drug, if it is a known effect of the drug and if it is unlikely or significantly less likely to be due to another cause.
2. **Possible**: an AE can be classified as possibly related to the medication if its timing is clearly related to the administration of the study drug, and the alternative cause is as or less likely than the possible relation to the study drug.
3. **Unlikely**: the relationship between an AE and the drug is unlikely if its timing is not related to the administration of the drug and/or there is a more likely alternative cause.
4. **Unrelated**: an AE is classified as unrelated to the study medication if it is due to an underlying or concurrent condition or it is the effect of another drug and its timing is not related to the administration of the study drug.

When it is difficult to classify an AE the investigator's opinion will prevail.

11.3. Intensity Classification

Intensity will be defined as follows:

- **Mild**: there is some discomfort which does not interfere with daily activities.
- **Moderate**: sufficient discomfort to reduce or affect daily activities.
- **Intense**: inability to work or perform daily activities.

11.4. Serious and Unexpected Adverse Events

A serious adverse event (SAE) or reaction is any untoward medical occurrence that at any dose:

- *results in death,*
- *is life-threatening,*

- *requires inpatient hospitalization or prolongation of existing hospitalisation,*
- *results in persistent or significant disability/incapacity, or*
- *is a congenital anomaly/birth defect.*

NOTE*:* The term "life-threatening" in the definition of "serious" refers to an event in which the patient was at risk of death at the time of the event; it does not refer to an event which hypothetically might have caused death if it were more severe.

Medical and scientific judgement should be exercised in deciding whether expedited reporting is appropriate in other situations, such as important medical events that may not be immediately life-threatening or result in death or hospitalisation but may jeopardise the patient or may require intervention to prevent one of the other outcomes listed in the definition above. *These should also usually be considered serious.*

Examples of such events are intensive treatment in an emergency room or at home for allergic bronchospasm; blood dyscrasias or convulsions that do not result in hospitalisation; or development of drug dependency or drug abuse.

An unexpected adverse event (UAE) is an adverse reaction which nature or severity is not consistent with the applicable product information (e.g., Investigator´s Brochure for an unapproved investigational medicinal product).

11.5. Serious adverse event reporting procedures

All SAE should be reported to the monitor within 24 hours of onset by telephone, followed by a written confirmation within three days of telephone notification, according to the form included in Appendix B of the protocol.

The sponsor must notify the AEMPS, CEIC and CCAA involved in the trial all suspected and unexpected serious adverse reactions that could be related to the investigational drugs no later than 7 calendar days after the sponsor has first knowledge of it if fatal or life-threatening and no later than 15 calendar days if non fatal or non life-threatening. This notification will be made within the above mentioned period even if all the information required on the form is not available. The form should be completed within 8 days.

11.6. General and specific rules for the investigators

During the study, if a patient suffers from an illness or adverse reaction which, in itself or because it requires pharmacological treatment, could alter the availability of any of the

study drugs, or the administration of which is contraindicated, the volunteer will be excluded from the study, specifying the reason in detail. If the adverse reaction is mild or moderate, whether it requires treatment or not, and does not involve suspending the drug, it will be recorded in detail and the study will continue as planned.

12. ETHICS

This clinical trial will be performed following the study protocol, the principles stated in the last Helsinki Declaration (see Annex 1), the Good Clinical Practice (GCP) Guidelines. The study complies with Clinical Trial Spanish regulation (Real Decreto de Ensayos Clínicos 223/2004) that includes the European Directive 2001/20/CE relative to the Member States Disposition about appliance of GCP in the development of Clinical Trials for drugs of human use.

The study investigators agree, when signing the protocol, to follow all the described procedures to comply with GCP guidelines. In agreement with RD 223/2004, the sponsor will submit all the required documents to the corresponding Reference Ethics Committee, as well as to the Ethics Committees of the rest of the participating centres. The study will not start until approval by both the Reference Ethics Committee and the Spanish Medicines Agency has been received.

When there is a situation causing a deviation from the protocol, the deviation will only be for the subject concerned. The investigators present in such circumstance will completely document the deviation and the reason for it in the CRF. If the deviation is related to the inclusion/exclusion criteria, the investigators will contact the clinical monitor by telephone to inform him/her of the deviation.

Any protocol amendment must be submitted to the Ethics Committee for their evaluation and approval in agreement with local requirements, and to the Spanish Medicines Agency when necessary.

13. DATA HANDLING AND RECORD KEEPING

13.1. Documentation archive

There will be a document archive for all the data, which shall be preserved in their entirety on paper and in electronic format for 15 years after completion of the study. This file shall contain the following:

1. IEC approval of the protocol and informed consent form.
2. Copy of the approved consent form and protocol, including any possible amendments.
3. All correspondence with the sponsor (Laboratorios Biomendi) related to and during the study.
4. All correspondence with the IEC.
5. Signed acceptance of the protocol.
6. *Curriculum vitae* of the principal investigator and the other members of the team of investigators.
7. Record of the signatures of the members of the team of investigators.
8. SAE reports.
9. Contract between the sponsor and the team of investigators.
10. Volunteer identity list.
11. Copies of the CRFs.

The documentation will be filed according to the CT unit's standard operating procedures (SOPs).

13.2. Medication management

The study medication will be provided by the sponsor and labelled according to the recommendations in Spanish regulation (RD 223/2004). In each centre, the Pharmacy centre will receive medication and store it until requested by an investigator.

14. FINANCING AND INSURANCE

14.1. Financing

Patients participating in the study will not receive any economic compensation. The financial compensation to be paid to investigators and the quiantity of the contract signed with the participating centres is specified in the financial summary.

14.2. Insurance

In accordance with Spanish legislation (Real Decreto 223/2004 of 6 February), Laboratorios Biomendi has taken out a civil liability insurance policy to cover possible

adversities derived from the study medication which could affect the subjects included in the trial.

15. CONFIDENTIALITY AND DATA PROTECTION

Written or oral information related to the study protocol, CRF or Investigator Brochure must be considered as confidential and is owned by the Sponsor of the study, Such information cannot be divulgated partially or totally by the principal Investigator or collaborators to a non authorized person without the formal Informed Consent of the Sponsor.

Investigators must warrant confidentiality about information regarding study patients or procedures. Every person participating in the study must consider this information as confidential as a means to keep personal and family intimacy of the participants. Treatment of sensible data will be performed in accordance with Spanish regulation (Ley Orgánica 16/99 de Protección de datos de carácter personal and RD 1720/2007, by which the regulation for development of Ley 15/1999, 13th December or Personal Data Protection is approved)

16. PUBLICATION POLICY

The principal investigator and/or the HOSPITAL, commit themselves not to use neither transmit to third persons, and not to disclose or to publish the results obtained in this trial without the previous sponsor written consent. The sponsor must have a copy of all manuscripts intended for publication thirty days before they are sent to the publisher.

17. REFERENCES

1. Southworth S, Peters J, Rock A, Pavliv L. A multicenter, randomized, double-blind, placebo-controlled trial of intravenous ibuprofen 400 and 800 mg every 6 hours in the management of postoperative pain. Clin Ther. 2009 Sep;31(9):1922-1935.

2. Singla N, Rock A, Pavliv L. A multi-center, randomized, double-blind placebo-controlled trial of intravenous-ibuprofen (IV-ibuprofen) for treatment of pain in post-operative orthopedic adult patients. Pain Med. 2010 Ago;11(8):1284-1293.

3. Kroll PB, Meadows L, Rock A, Pavliv L. A multicenter, randomized, double-blind, placebo-controlled trial of intravenous ibuprofen (i.v.-ibuprofen) in the management of postoperative pain following abdominal hysterectomy. Pain Pract. 2011 Feb;11(1):23-32.

4. Rathmell JP, Wu CL, Sinatra RS, Ballantyne JC, Ginsberg B, Gordon DB, et al. Acute post-surgical pain management: a critical appraisal of current practice, December 2-4, 2005. Reg Anesth Pain Med. 2006 Ago;31(4 Suppl 1):1-42.

5. LT. Intensive Care anethesia and analgesia. En: Current Diagnosis and Treatment: Critical Care. NY: McGraw Hill; 2008. p. 97-116.

6. Cancer, pain relief and palliative care. World Health Organization. 1990;

7. Vane JR, Botting RM. New insights into the mode of action of anti-inflammatory drugs. Inflamm. Res. 1995 Ene;44(1):1-10.

8. Marik PE. Fever in the ICU. Chest. 2000 Mar;117(3):855-869.

9. TORADOL, Ficah técnica del Producto.

10. Arora S, Wagner JG, Herbert M. Myth: parenteral ketorolac provides more effective analgesia than oral ibuprofen. CJEM. 2007 Ene;9(1):30-32.

11. Lloyd R, Derry S, Moore RA, McQuay HJ. Intravenous or intramuscular parecoxib for acute postoperative pain in adults. Cochrane Database Syst Rev. 2009;(2):CD004771.

12. Cadolor, Ficha Técnica del Producto.

13. Kauffman RE, Nelson MV. Effect of age on ibuprofen pharmacokinetics and antipyretic response. J. Pediatr. 1992 Dic;121(6):969-973.

14. Brown RD, Wilson JT, Kearns GL, Eichler VF, Johnson VA, Bertrand KM. Single-dose pharmacokinetics of ibuprofen and acetaminophen in febrile children. J Clin Pharmacol. 1992 Mar;32(3):231-241.

15. Kelley MT, Walson PD, Edge JH, Cox S, Mortensen ME. Pharmacokinetics and pharmacodynamics of ibuprofen isomers and acetaminophen in febrile children. Clin. Pharmacol. Ther. 1992 Ago;52(2):181-189.

16. Albert KS, Gillespie WR, Wagner JG, Pau A, Lockwood GF. Effects of age on the clinical pharmacokinetics of ibuprofen. Am. J. Med. 1984 Jul 13;77(1A):47-50.

17. Walson PD, Mortensen ME. Pharmacokinetics of common analgesics, anti-inflammatories and antipyretics in children. Clin Pharmacokinet. 1989;17 Suppl 1:116-137.

18. Morris PJ, Guntapalli K, Wright P, Bernard G. A multi-center, randomized, double-blind, placebo-controlled trial of the efficacy and safety of intravenous ibuprofen in febrile adults. Honolulu Hl: 2008.

19. Pavliv LR. A randomized, double-blind, placebo-controlled, single dose, crossover study of the pharmacokinetics, safety and tolerability of ibuprofen injection in healthy adult volunteers. Las Vegas, NV: 2009.

20. Ibuprofen. En: MartinDale. The Complete Drug Reference. 2009.

18. APPENDIX

18.1. Declaration of Helsinki

WORLD MEDICAL ASSOCIATION DECLARATION OF HELSINKI
Ethical Principles for Medical Research Involving Human Subjects

Adopted by the 18th WMA General Assembly, Helsinki, Finland, June 1964, and amended by the:
29th WMA General Assembly, Tokyo, Japan, October 1975
35th WMA General Assembly, Venice, Italy, October 1983
41st WMA General Assembly, Hong Kong, September 1989
48th WMA General Assembly, Somerset West, Republic of South Africa, October 1996
52nd WMA General Assembly, Edinburgh, Scotland, October 2000
53th WMA General Assembly, Washington 2002 (Note of Clarification on paragraph 29 added)
55th WMA General Assembly, Tokyo 2004 (Note of Clarification on Paragraph 30 added)
59th WMA General Assembly, Seoul, October 2008

A. INTRODUCTION

1. The World Medical Association (WMA) has developed the Declaration of Helsinki as a statement of ethical principles for medical research involving human subjects, including research on identifiable human material and data.
The Declaration is intended to be read as a whole and each of its constituent paragraphs should not be applied without consideration of all other relevant paragraphs.
2. Although the Declaration is addressed primarily to physicians, the WMA encourages other participants in medical research involving human subjects to adopt these principles.
3. It is the duty of the physician to promote and safeguard the health of patients, including those who are involved in medical research. The physician's knowledge and conscience are dedicated to the fulfilment of this duty.
4. The Declaration of Geneva of the WMA binds the physician with the words, "The health of my patient will be my first consideration," and the International Code of Medical Ethics declares that, "A physician shall act in the patient's best interest when providing medical care."
5. Medical progress is based on research that ultimately must include studies involving human subjects. Populations that are underrepresented in medical research should be provided appropriate access to participation in research.
6. In medical research involving human subjects, the well-being of the individual research subject must take precedence over all other interests.
7. The primary purpose of medical research involving human subjects is to understand the causes, development and effects of diseases and improve preventive, diagnostic and therapeutic interventions (methods, procedures and treatments). Even the best current interventions must be evaluated continually through research for their safety, effectiveness, efficiency, accessibility and quality.
8. In medical practice and in medical research, most interventions involve risks and burdens.
9. Medical research is subject to ethical standards that promote respect for all human subjects and protect their health and rights. Some research populations are particularly vulnerable and need special protection. These include those who cannot give or refuse consent for themselves and those who may be vulnerable to coercion or undue influence.
10. Physicians should consider the ethical, legal and regulatory norms and standards for research involving human subjects in their own countries as well as applicable international norms and standards. No national or international ethical, legal or regulatory requirement should reduce or eliminate any of the protections for research subjects set forth in this Declaration.

B. BASIC PRINCIPLES FOR ALL MEDICAL RESEARCH

11. It is the duty of physicians who participate in medical research to protect the life, health, dignity, integrity, right to self-determination, privacy, and confidentiality of personal information of research subjects.

12.Medical research involving human subjects must conform to generally accepted scientific principles, be based on a thorough knowledge of the scientific literature, other relevant sources of information, and adequate laboratory and, as appropriate, animal experimentation. The welfare of animals used for research must be respected.
13.Appropriate caution must be exercised in the conduct of medical research that may harm the environment.
14.The design and performance of each research study involving human subjects must be clearly described in a research protocol. The protocol should contain a statement of the ethical considerations involved and should indicate how the principles in this Declaration have been addressed. The protocol should include information regarding funding, sponsors, institutional affiliations, other potential conflicts of interest, incentives for subjects and provisions for treating and/or compensating subjects who are harmed as a consequence of participation in the research study. The protocol should describe arrangements for post-study access by study subjects to interventions identified as beneficial in the study or access to other appropriate care or benefits.
15.The research protocol must be submitted for consideration, comment, guidance and approval to a research ethics committee before the study begins. This committee must be independent of the researcher, the sponsor and any other undue influence. It must take into consideration the laws and regulations of the country or countries in which the research is to be performed as well as applicable international norms and standards but these must not be allowed to reduce or eliminate any of the protections for research subjects set forth in this Declaration. The committee must have the right to monitor ongoing studies. The researcher must provide monitoring information to the committee, especially information about any serious adverse events. No change to the protocol may be made without consideration and approval by the committee.
16.Medical research involving human subjects must be conducted only by individuals with the appropriate scientific training and qualifications. Research on patients or healthy volunteers requires the supervision of a competent and appropriately qualified physician or other health care professional. The responsibility for the protection of research subjects must always rest with the physician or other health care professional and never the research subjects, even though they have given consent.
17.Medical research involving a disadvantaged or vulnerable population or community is only justified if the research is responsive to the health needs and priorities of this population or community and if there is a reasonable likelihood that this population or community stands to benefit from the results of the research.
18.Every medical research study involving human subjects must be preceded by careful assessment of predictable risks and burdens to the individuals and communities involved in the research in comparison with foreseeable benefits to them and to other individuals or communities affected by the condition under investigation.
19.Every clinical trial must be registered in a publicly accessible database before recruitment of the first subject.
20.Physicians may not participate in a research study involving human subjects unless they are confident that the risks involved have been adequately assessed and can be satisfactorily managed. Physicians must immediately stop a study when the risks are found to outweigh the potential benefits or when there is conclusive proof of positive and beneficial results.
21.Medical research involving human subjects may only be conducted if the importance of the objective outweighs the inherent risks and burdens to the research subjects.
22.Participation by competent individuals as subjects in medical research must be voluntary. Although it may be appropriate to consult family members or community leaders, no competent individual may be enrolled in a research study unless he or she freely agrees.
23.Every precaution must be taken to protect the privacy of research subjects and the confidentiality of their personal information and to minimize the impact of the study on their physical, mental and social integrity.
24.In medical research involving competent human subjects, each potential subject must be adequately informed of the aims, methods, sources of funding, any possible conflicts of interest, institutional affiliations of the researcher, the anticipated benefits and potential risks of the study and the discomfort it may entail, and any other relevant aspects of the study. The potential subject must be informed of the right to refuse to participate in the study or to withdraw consent to participate at any time without reprisal. Special attention should be given to the specific information needs of individual

potential subjects as well as to the methods used to deliver the information. After ensuring that the potential subject has understood the information, the physician or another appropriately qualified individual must then seek the potential subject's freely-given informed consent, preferably in writing. If the consent cannot be expressed in writing, the non-written consent must be formally documented and witnessed.

25. For medical research using identifiable human material or data, physicians must normally seek consent for the collection, analysis, storage and/or reuse. There may be situations where consent would be impossible or impractical to obtain for such research or would pose a threat to the validity of the research. In such situations the research may be done only after consideration and approval of a research ethics committee.

26. When seeking informed consent for participation in a research study the physician should be particularly cautious if the potential subject is in a dependent relationship with the physician or may consent under duress. In such situations the informed consent should be sought by an appropriately qualified individual who is completely independent of this relationship.

27. For a potential research subject who is incompetent, the physician must seek informed consent from the legally authorized representative. These individuals must not be included in a research study that has no likelihood of benefit for them unless it is intended to promote the health of the population represented by the potential subject, the research cannot instead be performed with competent persons, and the research entails only minimal risk and minimal burden.

28. When a potential research subject who is deemed incompetent is able to give assent to decisions about participation in research, the physician must seek that assent in addition to the consent of the legally authorized representative. The potential subject's dissent should be respected.

29. Research involving subjects who are physically or mentally incapable of giving consent, for example, unconscious patients, may be done only if the physical or mental condition that prevents giving informed consent is a necessary characteristic of the research population. In such circumstances the physician should seek informed consent from the legally authorized representative. If no such representative is available and if the research cannot be delayed, the study may proceed without informed consent provided that the specific reasons for involving subjects with a condition that renders them unable to give informed consent have been stated in the research protocol and the study has been approved by a research ethics committee. Consent to remain in the research should be obtained as soon as possible from the subject or a legally authorized representative.

30. Authors, editors and publishers all have ethical obligations with regard to the publication of the results of research. Authors have a duty to make publicly available the results of their research on human subjects and are accountable for the completeness and accuracy of their reports. They should adhere to accepted guidelines for ethical reporting. Negative and inconclusive as well as positive results should be published or otherwise made publicly available. Sources of funding, institutional affiliations and conflicts of interest should be declared in the publication. Reports of research not in accordance with the principles of this Declaration should not be accepted for publication.

C. ADDITIONAL PRINCIPLES FOR MEDICAL RESEARCH COMBINED WITH MEDICAL CARE

31. The physician may combine medical research with medical care only to the extent that the research is justified by its potential preventive, diagnostic or therapeutic value and if the physician has good reason to believe that participation in the research study will not adversely affect the health of the patients who serve as research subjects.

32. The benefits, risks, burdens and effectiveness of a new intervention must be tested against those of the best current proven intervention, except in the following circumstances:

- The use of placebo, or no treatment, is acceptable in studies where no current proven intervention exists; or
- Where for compelling and scientifically sound methodological reasons the use of placebo is necessary to determine the efficacy or safety of an intervention and the patients who receive placebo or no treatment will not be subject to any risk of serious or irreversible harm. Extreme care must be taken to avoid abuse of this option.

33. At the conclusion of the study, patients entered into the study are entitled to be informed about the outcome of the study and to share any benefits that result from it, for example, access to interventions identified as beneficial in the study or to other appropriate care or benefits.

34. The physician must fully inform the patient which aspects of the care are related to the research. The refusal of a patient to participate in a study or the patient's decision to withdraw from the study must never interfere with the patient-physician relationship.
35. In the treatment of a patient, where proven interventions do not exist or have been ineffective, the physician, after seeking expert advice, with informed consent from the patient or a legally authorized representative, may use an unproven intervention if in the physician's judgement it offers hope of saving life, re-establishing health or alleviating suffering. Where possible, this intervention should be made the object of research, designed to evaluate its safety and efficacy. In all cases, new information should be recorded and, where appropriate, made publicly available.

22.10.2008

18.2. Serious or Unexpected Adverse Events Spanish Report Form

INSTRUCCIONES GENERALES

1. Este formulario se utilizará solamente para comunicar las sospechas de reacciones adversas (RA) graves e inesperadas que ocurran con medicamentos en investigación. Se considera medicamento en investigación tanto el producto específicamente investigado como el control.

2. Las sospechas de reacciones adversas mortales o que entrañen riesgo vital (aquellas que de no haber mediado una intervención terapéutica inmediata hubieran supuesto la muerte del paciente) se comunicarán en el plazo máximo de 7 días naturales; si no se dispusiera de toda la información, ésta podrá completarse en el plazo adicional de 8 días. Las demás sospechas de reacciones adversas graves e inesperadas se comunicarán en el plazo máximo de 15 días.

3. Cuando el espacio disponible sea insuficiente, se añadirá una hoja de información adicional, correctamente identificada con el nombre del promotor y el número asignado a la notificación. En esta información adicional podrá hacerse constar la evaluación de la causalidad realizada por el técnico que informa.

INSTRUCCIONES ESPECÍFICAS

1. El código de protocolo es el asignado por el promotor para identificar el ensayo. El número de notificación del promotor es el que éste utiliza para su archivo. Cuando se trate de información de seguimiento se utilizará el mismo número o bien, si se modifica, se indicará el número de la notificación inicial. Se dejará sin rellenar el espacio “Nº de notificación” que aparece sombreado.

2. La edad se pondrá en años, meses, semanas o días según convenga, pero siempre indicándolo. Si no se conoce con precisión la edad debe referirse, al menos, el grupo de edad al que pertenece (p. ej.: lactante, niño, adolescente, adulto, anciano).

7. Se describirá la reacción adversa en forma completa, indicando la fecha de finalización de la misma e incluyendo los resultados de las exploraciones complementarias o pruebas de laboratorio que se consideren de interés. A esta notificación podrán acompañarse cuantos informes se estimen convenientes para la adecuada interpretación del cuadro clínico sospechoso de ser una reacción adversa.

8-13. Las categorías no son mutuamente excluyentes. La asistencia en un Servicio de Urgencias de un Hospital inferior a 24 horas, no se considerará hospitalización.

14. Los medicamentos en investigación se identificarán a ser posible por su nombre genérico (DOE o DCI), indicando cuando esté disponible el nombre comercial, o en su defecto, por el nombre propuesto o código de laboratorio para el producto.

15. En caso de que la administración no sea diaria se intentará describirla con alguna de las siguientes posibilidades: cíclica, semanal, mensual, anual o número de veces que se ha utilizado (poniendo en este caso la dosis de cada toma, no la total).

17. Se hará constar el proceso patológico del paciente al que va destinado el producto en investigación, o bien “voluntario sano” en caso de tratarse de tal.

19. Se hará constar la duración del tratamiento hasta el inicio de la reacción adversa.

22. Se indicará explícitamente si no se han tomado fármacos concomitantes. En el caso de considerar sospechoso alguno o algunos de los fármacos concomitantes se marcarán con un asterisco (p.ej.: * AMOXICILINA). Se excluirán los medicamentos utilizados para tratar la reacción adversa.

FORMULARIO DE NOTIFICACIÓN DE RAGI OCURRIDA EN ESPAÑA

NOTIFICACION DE SOSPECHA DE REACCION ADVERSA PARA **MEDICAMENTOS EN INVESTIGACIÓN**	CODIGO DE PROTOCOLO (promotor)	Nº NOTIFICACION (Promotor)
	PACIENTE Nº	Nº NOTIFICACION

I. INFORMACION SOBRE LA REACCIÓN ADVERSA

1a. PAÍS	2. FECHA DE NACIMIENTO			2a. EDAD	3. SEXO	3a. PESO	3b. TALLA	4-6. FECHA DE INICIO DE LA REACCIÓN		
	DÍA	MES	AÑO		☐ HOMBRE<br>☐ MUJER			DÍA	MES	AÑO

7. DESCRIPCIÓN DE LA REACCIÓN ADVERSA (Incluyendo resultados relevantes de exploración o de laboratorio, y la fecha de finalización, si procede).	8-13b. CRITERIOS DE GRAVEDAD/ DESENLACE<br>☐ FALLECIMIENTO<br>☐ LA VIDA DEL PACIENTE HA ESTADO EN PELIGRO<br>☐ HOSPITALIZACIÓN<br>☐ PROLONGACIÓN HOSPITALIZACIÓN<br>☐ INCAPACIDAD PERMANENTE O SIGNIFICATIVA<br>☐ RA CLINICAMENTE RELEVANTE<br>☐ PERSISTENCIA DE LA REACCIÓN ADVERSA<br>☐ RECUPERACIÓN

II. INFORMACION DEL MEDICAMENTO EN INVESTIGACIÓN

14. MEDICAMENTO SOSPECHOSO	15. DOSIS DIARIA	16. VÍA	17. ENFERMEDAD EN ESTUDIO	18. FECHAS DE INICIO	FINAL	19. DURACIÓN DEL TRATAMIENTO

20. ¿REMITIÓ LA REACCIÓN AL SUSPENDER LA MEDICACIÓN?	20a. ¿REMITIÓ LA REACCIÓN AL REDUCIR LA DOSIS?	21. ¿REAPARECIÓ LA REACCIÓN AL ADMINISTRAR DE NUEVO LA MEDICACIÓN?
☐ SI ☐ NO ☐ NO PROCEDE	☐ SI ☐ NO ☐ NO PROCEDE	☐ SI ☐ NO ☐ NO PROCEDE

III. MEDICAMENTOS CONCOMITANTES E HISTORIA CLÍNICA

22. MEDICAMENTOS CONCOMITANTES (Márquese con un asterisco el o los medicamentos sospechosos)	22a. DOSIS DIARIA	22b. VÍA	22c. FECHAS DE INICIO	FINAL	22d. MOTIVO DE LA PRESCRIPCIÓN

23. DATOS IMPORTANTES DE LA HISTORIA CLÍNICA (ej. diagnósticos, alergias, embarazos, etc.)

IV. INFORMACION SOBRE PROMOTOR E INVESTIGADOR

24a. NOMBRE Y DIRECCION DEL PROMOTOR		24b. NOMBRE Y DIRECCION DEL INVESTIGADOR
24c. CODIGO DE LABORATORIO<br>(Nº AEM)	25a. TIPO DE INFORME<br>YINICIAL<br>YSEGUIMIENTO	24c. TECNICO DEL PROMOTOR QUE INFORMA<br>NOMBRE:<br>TELEFONO:<br>FIRMA:
24e. FECHA DEL INFORME	24f. FECHA DE ENTRADA AEM	25b. SE ADJUNTA INFORME COMPLEMENTARIO

18.3. Patient Information Sheet and Informed Consent form

TITULO DEL ESTUDIO: "ESTUDIO MULTICÉNTRICO, ALEATORIZADO, DOBLE CIEGO, CONTROLADO CON PLACEBO, PARA EVALUAR LA EFICACIA Y SEGURIDAD DE UNA NUEVA FORMULACIÓN DE IBUPROFENO 800 MG CADA 6 HORAS EN EL MANEJO DEL DOLOR POSTOPERATORIO"

Promotor: Laboratorios Biomendi S.A.U"

Código del estudio: BIBEC02
Código EudraCT: 2011-005007-33
Unidad del Dolor
Servicio de Farmacología Clínica
Servicio de Anestesiología
Urgencias. Sección de Farmacología Clínica
Hospital General Universitario de la Princesa

Coordinadores del estudio: **Concepción Pérez Hernández**
Francisco Abad Santos
Dolores Ochoa Mazarro
Antonio Planas
Hospital General Universitario de la Princesa

Introducción

En los próximos días, usted va a ser intervenido de una cirugía traumatológica (prótesis de cadera o de rodilla) o abdominal (hernia inguinal, extirpación de la vesícula, extirpación del apéndice) tras la cual se precisa administrar medicación analgésica con el fin de evitar que usted tenga dolor. Por ello, se le propone que participe en el ensayo de investigación clínica titulado "Estudio multicéntrico, aleatorizado, doble ciego, controlado con placebo, para evaluar la eficacia y seguridad de ibuprofeno intravenoso 800 mg cada 6 horas en el manejo del dolor postoperatorio". El estudio ha sido aprobado por el Comité Ético de Investigación Clínica correspondiente y por la Agencia Española del Medicamento y Productos Sanitarios, de acuerdo con la legislación vigente, el RD 223/2004, de 6 de Febrero por el que se regulan los ensayos clínicos con medicamentos.

Para decidir su participación en él, usted debe comprender la finalidad del estudio y el modo en que el tratamiento puede beneficiarle, conociendo los riesgos que puede entrañar su participación en el mismo. La información que usted necesita se encuentra en esta *Hoja de Información* que se le proporciona para que lea con detenimiento. Si después de haberla leído y haber aclarado sus dudas con el personal investigador desea participar, se le solicitará que firme el Consentimiento Informado y se le proporcionará una copia del mismo.

PARTICIPACIÓN VOLUNTARIA

Debe saber que su participación es voluntaria y que puede decidir no participar o cambiar su decisión y retirar el consentimiento en cualquier momento, sin que por ello se altere la relación con su médico ni se produzca perjuicio alguno en su tratamiento.

DESCRIPCIÓN Y OBJETIVOS DEL ESTUDIO

Ibuprofeno es un fármaco perteneciente al grupo de los antiinflamatorios no esteroideos que habitualmente se utiliza por vía oral para el tratamiento del dolor agudo o crónico, así como para el control de la fiebre. A pesar de que es un fármaco de uso muy común por vía oral, hasta el momento no se dispone en nuestro país de una formulación para utilizarlo por vía intravenosa en aquellas situaciones en las que no se puede utilizar medicación por vía oral, tales como el manejo del dolor agudo tras una cirugía. En estos casos se utilizan otros antiinflamatorios que sí están disponibles para su administración intravenosa, como el ketorolaco (cuyo nombre comercial es

Toradol) o el desketoprofeno (cuyo nombre comercial es Enantyum). Además de estos fármacos antiinflamatorios, tras la cirugía se administra habitualmente otro tipo de fármacos analgésicos como son los opioides mayores para el control del dolor.

El promotor de este estudio, Laboratorios Biomendi, está desarrollando una formulación intravenosa de ibuprofeno para ser comercializada en España, y el objetivo de este estudio es estudiar la eficacia y seguridad de su administración para el manejo del dolor agudo postoperatorio.

Este estudio tiene un diseño aleatorizado, doble ciego y controlado con placebo. Un estudio **aleatorizado** significa que en él, la asignación del paciente a uno u otro grupo de tratamiento se realiza al azar, como si para decidirlo tiráramos una moneda al aire. **Doble ciego** significa que ni usted ni el investigador que evaluará su respuesta al fármaco sabrán qué tratamiento se le está administrando. **Controlado con placebo** significa que en uno de los dos grupos de tratamiento se va a administrar Placebo, es decir, una sustancia farmacológicamente inactiva. No obstante, esto no quiere decir que usted no vaya a recibir ninguna medicación para el dolor. El protocolo de estudio contempla que a usted se le administre morfina para el control del dolor como se hace de forma habitual. Además, si con esto no fuera suficiente, usted puede solicitar la administración de medicación de rescate (diferente de la planificada en el estudio) para el control de su dolor.

En caso de que usted haya decidido dar su consentimiento para participar en el estudio y tras comprobar que cumple con los requisitos del mismo le asignaremos al azar (igual que si lanzáramos una moneda al aire) a unos de los siguientes grupos de tratamiento:

- Grupo de tratamiento activo, en el que recibirá 800 mg de ibuprofeno por vía intravenosa cada 6 horas durante un máximo 3 días (24 h en caso de cirugía abdominal, 48 en cirugía de cadera y 72 h en cirugía de rodilla). La administración del fármaco empezará en el momento de cierre de la herida quirúrgica. La infusión de cada dosis durará 15 minutos.
- Grupo de placebo, en el que recibirá 200 ml de suero salino fisiológico cada 6 horas durante un máximo de 3 días (24 h en caso de cirugía abdominal, 48 en cirugía de cadera y 72 h en cirugía de rodilla).La administración del fármaco empezará en el momento de cierre de la sutura de la herida quirúrgica. La infusión de cada dosis durará 15 minutos.

Además de recibir el tratamiento al que haya sido asignado, usted recibirá morfina de acuerdo con la práctica clínica habitual del centro donde se encuentre.

Este estudio se va a llevar a cabo en 12 hospitales españoles y se planifica incluir un total de 160 pacientes como usted en cada grupo de tratamiento (320 pacientes en total).

Como se ha descrito más arriba, la administración de la medicación del estudio comenzará 30 minutos después de la sutura de la herida quirúrgica. Antes de la infusión de la primera dosis y tras 1, 3, 6, 12, 18, 24, 32, 40, 48, 56, 64 y 72 horas se le solicitará que cuantifique la intensidad del dolor que padece(si se intrumpe el tratamiento con ibuprofeno solo se medirá hasta 6 horas de la última dosis). En cada uno de estos momentos se recogerá también la cantidad de morfina que ha precisado utilizar para el control de su dolor. Además, se extraerán de su historia clínica las variables relevantes para el estudio: constantes vitales (tensión arterial, pulsaciones, frecuencia respiratoria, temperatura), determinaciones de laboratorio, etc. Tras esto, se iniciará un período de seguimiento durará hasta 1 semana después del inicio de la medicación. Asimismo, durante todo el estudio se le preguntará sobre posibles efectos adversos que usted pudiera estar padeciendo.

Por lo demás, recibirá los otros tratamientos y cuidados indicados en pacientes como usted, y se le realizarán las pruebas complementarias habituales y aquellas que su médico considere necesarias.

Usted puede dejar de participar en el estudio en cualquier momento y sin que ello altere la relación con su médico ni el tratamiento que se le va a ofrecer. Además, a criterio del investigador usted podrá ser retirado del estudio si sucede alguno de los siguientes:

- Que haya precisado la administración de medicación analgésica diferente del fármaco en estudio o de la morfina contemplada en el protocolo.

- Que usted ya tolere medicación por vía oral.
- Que el dolor desaparezca.
- Que se pierda el acceso venoso por donde se infunde la medicación.
- Que usted sea dado de alta.
- Que usted no cumpla adecuadamente con los procedimientos del estudio.

Una vez retirado del estudio, ya sea por su propio criterio o por decisión del investigador, no se le administrarán más dosis de la medicación en estudio, pero se le realizará la visita de seguimiento correspondiente a los 3 días de la última dosis (si ha sido dado de alta simplemente se le llamará por teléfono para comprobar que usted se encuentra bien).

POSIBLES ACONTECIMIENTOS ADVERSOS

Ibuprofeno es un fármaco por lo general bien tolerado. Las reacciones adversas más frecuentemente comunicadas son náuseas, flatulencia, vómitos y dolor de cabeza. Ibuprofeno pertenece al grupo de fármacos antiinflamatorios no esteroideos (conocidos por las siglas AINE). Ibuprofeno, como otros AINE, puede causar eventos cardiovasculares graves tales como infarto de miocardio o accidente cerebrovascular. Además, puede causar alteraciones gastrointestinales desde leves (las más frecuentes, principalmente ardor de estómago y malestar abdominal) hasta graves (menos frecuentes, como hemorragias digestivas o úlceras gástricas). También puede causar alteraciones de la función hepática que van desde alteraciones en los parámetros analíticos (lo más frecuente) hasta cuadros de hepatitis agudas (más raramente). De forma rara puede causar alteraciones dermatológicas graves y reacciones alérgicas.

POLIZA DE SEGURO

De acuerdo con el RD de Ensayos Clínicos 223/2004, de 6 de Febrero por el que se regulan los ensayos clínicos con medicamentos, la realización de este estudio requiere la contratación de un seguro de Responsabilidad Civil para dar cobertura a posibles reacciones adversas que pudieran aparecer durante el desarrollo del estudio. Laboratorios Biomendi ha contratado con la Compañía ***** tal seguro, con número de póliza ******.

No obstante, cualquier nueva información que aparezca en el curso del estudio sobre este fármaco se le comunicará lo antes posible.

BENEFICIOS ESPERADOS

Es posible que usted no se beneficie directamente de la participación en este estudio; no obstante, la información que se obtenga servirá para conocer la eficacia y seguridad de la formulación intravenosa de ibuprofeno y para su eventual comercialización.

CONFIDENCIALIDAD DE LOS DATOS

Toda la información clínica que se obtenga referente a sus antecedentes médicos y a su enfermedad actual se tratará en todo momento de manera confidencial y anónima, identificando los datos mediante un código y no por su nombre ni cualquier otro identificador que le relacione con esta información. En todo momento se respetará la normativa sobre el tratamiento de datos personales regulada por la Ley 15/1999 de Protección de Datos de Carácter Personal.

Este estudio es un ensayo clínico promovida por Laboratorios Biomendi con el objetivo de estudiar la eficacia y seguridad de la formulación de ibuprofeno intravenoso que se quiere comercializar. El promotor ha firmado con los centros en los que se prevé realizar el estudio un contrato, y los investigadores que participen en el mismo recibirán una compensación económica proporcional al trabajo desempeñado en el mismo.

Si tiene alguna duda sobre la información que acaba de leer, pregunte al médico que le ha facilitado esta hoja. Sobre cualquier duda o problema relacionado con el estudio puede contactar con los investigadores coordinadores del estudio o alguno de sus colaboradores

Coordinadores del estudio

Concepción Pérez Hernández (1), Francisco Abad Santos (2), Dolores Ochoa Mazarro (2), Antonio Planas (3). 1 Unidad del Dolor. Servicio de Anestesiología. Pain Unit. Anesthesiology Department, 2 Servicio de Farmacología Clínica, 3 Servicio de Anestesiología

Hospital Universitario de la Princesa

Diego de León 62

28006 MADRID, SPAIN.

Phone: +34 91 520 24 25.

Fax: +34 91 520 25 40

Nombre del participante: ______________________________

Firma y fecha:

Nombre del investigador: ______________________________

Firma y fecha:

CONSENTIMIENTO INFORMADO POR ESCRITO PARA EL PACIENTE

TITULO DEL ESTUDIO: "ESTUDIO MULTICÉNTRICO, ALEATORIZADO, DOBLE CIEGO, CONTROLADO CON PLACEBO, PARA EVALUAR LA EFICACIA Y SEGURIDAD DE UNA NUEVA FORMULACIÓN DE IBUPROFENO 800 MG CADA 6 HORAS EN EL MANEJO DEL DOLOR POSTOPERATORIO"

Promotor: Laboratorios Biomendi S.A.U"

Código del estudio: BIBEC02
Código EudraCT: 2011-005007-33
Unidad del Dolor
Servicio de Farmacología Clínica
Servicio de Anestesiología
Urgencias. Sección de Farmacología Clínica
Hospital General Universitario de la Princesa

Yo (Nombre y apellidos)
....

- He leído la hoja de información que se me ha entregado.
- He podido hacer preguntas sobre el estudio.
- He recibido suficiente información sobre el estudio.
- He hablado con ....
(Nombre y apellidos del investigador)

Comprendo que mi participación es voluntaria.

Comprendo que puedo retirarme del estudio:
- Cuando quiera.
- Sin tener que dar explicaciones.
- Sin que esto repercuta en mis cuidados médicos.

Presto libremente mi conformidad para participar en el estudio.

En,..a................. de.................................. de...........

Firma del participante Firma del Investigador
Fecha: Fecha:

CONSENTIMIENTO INFORMADO POR ESCRITO PARA EL FAMILIAR/ REPRESENTANTE LEGAL

TITULO DEL ESTUDIO: "ESTUDIO MULTICÉNTRICO, ALEATORIZADO, DOBLE CIEGO, CONTROLADO CON PLACEBO, PARA EVALUAR LA EFICACIA Y SEGURIDAD DE IBUPROFENO INTRAVENOSO 800 MG CADA 6 HORAS EN EL MANEJO DEL DOLOR POSTOPERATORIO"

Promotor: Laboratorios Biomendi S.A.U"

Código del estudio: BIBE-C02
Código EudraCT: 2011-005007-33
Unidad del Dolor
Servicio de Farmacología Clínica
Servicio de Anestesiología
Urgencias. Sección de Farmacología Clínica
Hospital General Universitario de la Princesa

Yo, (Nombre y apellidos)…………………………………………………en calidad de………………..
(Relación con el participante) de………………………………….. (nombre y apellidos del participante) de…………………………………………………….

- He leído la hoja de información que se me ha entregado.
- He podido hacer preguntas sobre el estudio.
- He recibido suficiente información sobre el estudio.
- He hablado con ....
(nombre y apellidos del investigador)

Comprendo que la participación del paciente es voluntaria.

Comprendo que puede retirarse del estudio:
- Cuando quiera.
- Sin tener que dar explicaciones.
- Sin que esto repercuta en sus cuidados médicos.

En mi presencia se ha dado a ……………………………………..(nombre del participante) toda la información pertinente adaptada a su nivel de entendimiento y está de acuerdo en participar. Presto mi conformidad para que……………………………………………(nombre del participante) participe en este estudio y doy mi consentimiento para el acceso y utilización de los datos en las condiciones detalladas en la hoja de información.
Presto libremente mi conformidad para que participe en el estudio.

Firma del representante Firma del Investigador
Fecha Fecha
