# Supplementary material for: Intravenous Ibuprofen for Treatment of Post-Operative Pain: A Multicenter, Double Blind, Placebo-Controlled, Randomized Clinical Trial
Source: PLoS One. 2016 May 6;11(5):e0154004. doi: 10.1371/journal.pone.0154004 (PMC4859493; doi:10.1371/journal.pone.0154004)
Supplement: S1 Table — (PDF) [file pone.0154004.s003.pdf]

S1 Table A. Serious Adverse Events

Study treatment	Adverse Event	MedDRA Preferred Term	NCI-CTCAE grade	Outcome	Action Taken
Ibuprofen[a]	Severe induration	Injection site induration	3	Resolved	None
Ibuprofen[a]	Respiratory infection	Respiratory tract infection	3	Resolved	None
Placebo[b]	Headache	Headache	3	Resolved	Permanently discontinued

Safety Population. All events were catalogued as unrelated to the study as regards to causality. [a] patient from abdominal surgery; [b] patient from orthopaedic surgery.
