# Supplementary material for: Intravenous Ibuprofen for Treatment of Post-Operative Pain: A Multicenter, Double Blind, Placebo-Controlled, Randomized Clinical Trial
Source: PLoS One. 2016 May 6;11(5):e0154004. doi: 10.1371/journal.pone.0154004 (PMC4859493; doi:10.1371/journal.pone.0154004)
Supplement: S2 Table — (PDF) [file pone.0154004.s004.pdf]

S2 Table B. Most frequent treatment emergent adverse events.

MedDRA Preferred Term	Ibuprofen (n=107)	Placebo (n=99)	P-Value
Pyrexia, n (%)	5 (3.57)	9 (9.09)	Chi-Square: 0.2081
Nausea, n (%)	8 (7.48)	5 (5.05)	Chi-Square: 0.4743
Vomiting, n (%)	4 (3.74)	2 (2.02)	Fisher: 0.6843
Urinary retention, n (%)	2 (1.87)	4 (4.04)	Fisher: 0.6729
Alanine aminotransferase increased, n (%)	2 (1.87)	3 (3.03)	Fisher: 0.4305
Gamma-glutamyltransferase increased, n (%)	1 (0.93)	2 (2.02)	Fisher: 0.6090

Most frequent are considered the ones which occurs in more than 2% of the Safety population.
