# Supplementary material for: Intravenous Ibuprofen for Treatment of Post-Operative Pain: A Multicenter, Double Blind, Placebo-Controlled, Randomized Clinical Trial
Source: PLoS One. 2016 May 6;11(5):e0154004. doi: 10.1371/journal.pone.0154004 (PMC4859493; doi:10.1371/journal.pone.0154004)
Supplement: S3 Table — (PDF) [file pone.0154004.s005.pdf]

S3 Table C. Summary of local reactions

	Ibuprofen [a] (n=107)	Placebo [b] (n=99)	P Value Test
**Pain**			
n (%)	63 (58.88)	51 (51.52)	Fisher: 0.3122
**Burning**			
n (%)	64 (59.81)	51 (51.52)	Fisher: 0.2472
**Erythema**			
n (%)	3 (2.80)	3 (3.03)	Fisher: 1.0000
**Induration**			
n (%)	3 (2.80)	0 (0.00)	Fisher: 0.2471

Local reactions reported at any time point during the study period (safety population). Patients that did not report these data are shown as Missing data from: [a] 5 patients, [b] 4 patients.
